# Supplementary material for: Conditional and interaction gene-set analysis reveals novel functional pathways for blood pressure
Source: Nat Commun. 2018 Sep 14;9:3768. doi: 10.1038/s41467-018-06022-6 (PMC6138636; doi:10.1038/s41467-018-06022-6)
Supplement: Supplementary file 1 — Supplementary Information [file 41467_2018_6022_MOESM1_ESM.pdf]

Supplementary information for

Conditional and interaction gene-set analysis reveals novel functional pathways for blood pressure

De Leeuw et al.

SUPPLEMENTARY METHODS

CONTENTS

UK BIOBANK DATA AND QC 3

ANALYSIS GUIDELINE 4

STEP 1: INITIAL GSA 4
STEP 2: CORRECTING FOR GENERAL CONFOUNDING 6
STEP 3: OVERLAPPING ASSOCIATIONS 7
STEP 4: DETECTING HIDDEN CONFOUNDING AND OUTLIERS 9
INTERACTION ANALYSIS 10
STEP 5: POST-HOC INTERACTION ANALYSIS 13
STEP 6: EXPLORATORY INTERACTION ANALYSIS 14

DETAILED OVERVIEW OF BLOOD PRESSURE ANALYSIS 15

STEP 1: INITIAL GSA 15
STEP 2: CORRECTING FOR GENERAL CONFOUNDING 15
STEP 3: OVERLAPPING ASSOCIATIONS 16
STEP 4: DETECTING HIDDEN CONFOUNDING AND OUTLIERS 19
STEP 5: POST-HOC INTERACTION ANALYSIS 20
STEP 6: EXPLORATORY INTERACTION ANALYSIS 22

SIMULATION STUDY 23

REPLICATION ANALYSIS 25

DATA 25
RESULTS 25

GENE-SET ANALYSES IN RECENT BLOOD PRESSURE GWAS 26

OVERVIEW 26
COMPARISON 26

SUPPLEMENTARY REFERENCES 28

SUPPLEMENTARY FIGURES 29

SUPPLEMENTARY TABLES 35

Note: Supplementary Tables 9 through 18 can be found in the Supplementary Data 1 file

UK Biobank data and QC

Genotype data from the UK Biobank[1] project (www.ukbiobank.ac.uk) was used for the analysis. All participants in the UK Biobank project have provided written informed consent. UK Biobank received ethical approval from the National Research Ethics Service Committee North West Haydock (reference 11/NW/0382), and all study procedures for the project were performed in accordance with the World Medical Association Declaration of Helsinki ethical principles for medical research. The current study was conducted under the UK Biobank application number 16406. The full study design of UK Biobank is described elsewhere[1].

Imputed genotype data from the July 2017 release of UK Biobank was used, and full details on the data are provided here: http://www.biorxiv.org/content/early/2017/07/20/166298. In brief, initial quality control of the markers and samples was performed by UK Biobank resulting in a data set containing 805,426 markers and 488,377 individuals. Genotype imputation was then performed using two reference panels: a merged reference panel consisting of UK10K haplotypes[2] and the 1,000 Genomes (phase 3)[3] reference panel; and the Haplotype Reference Consortium (HRC)[4] panel. For variants imputed in both panels, the HRC imputation was used. The downloaded BGEN data files used as input for our own additional QC contained a total of 97,059,329 autosomal variants and 487,409 individuals.

Additional processing and QC were performed on the imputed data. First, the imputed dosages were converted to hard-called genotypes using PLINK[5], at the default hard call threshold of 0.1. Variants were then filtered on missingness (missingness < 5%), minor allele frequency (MAF > 1e-6) and imputation quality (info score ≥ 0.9). After filtering, 17,366,833 variants remained in the data. Following the advice of UK Biobank we also removed imputed variants that were not imputed on the HRC panel, leaving 13,923,638 variants.

For filtering of individuals, the UK Biobank data was projected onto the first 30 principal components of the 1,000 Genomes (phase 3) data using SmartPCA[6]. Each individual in the UK Biobank data was then assigned to the subpopulation of the 1,000 Genomes individual closest to it, measured by the Mahalanobis distance. For individuals for which this distance was greater than six standard deviations the ancestry was set to missing; these were removed from the analysis, reducing the sample size by 834 individuals.

Individuals were then filtered on a number of quality control criteria. Based on metrics provided by UK Biobank, the following filtering was applied (in this order): mismatch between reported sex and genetic sex (372 individuals) or sex aneuploidy (471 individuals); and excluded in kinship inference (8 individuals) or excessive number of relatives (187 individuals). This left the sample size at 485,540 individuals.

The sample was then restricted to individuals of European ancestry only according to the 1,000 Genomes ancestry mapping, reducing it to 459,984 individuals. Filtering was then applied to obtain an independent sample, with no pairs of individuals with a kinship coefficient greater than 0.04 (kinship coefficients were provided by UK Biobank). To do so individuals were removed from the sample one at a time, every time removing the individual with the highest number of related other individuals in the sample.

This was continued until no pairs of individuals with kinship coefficient greater than 0.04 remained. Dropping an additional seven individuals still in the sample who had withdrawn consent brought the sample size to 387,112. Principal components where then computed using FlashPCA[7], to be included as covariates to correct for population structure. Finally, the sample was filtered on individuals with missing values on covariates or any of the phenotypes, for a final sample size of 360,243 individuals for the analyses.

ANALYSIS GUIDELINE

The aim of the extended GSA workflow is to provide more detailed information on the gene property associations found in an initial standard GSA, which can be used to refine interpretation of results. Conditional analyses can be used to detect confounding, and to evaluate the relations between the associations of overlapping or correlated gene properties. This helps avoid drawing misleading conclusions and allows association signals shared by multiple gene properties to be correctly understood. It can also be informative in providing evidence for the likely absence of confounding, and the relative independence of associations in gene properties that do to some extent correlate or overlap.

The interaction analyses can be used to refine associations, narrowing them down to subsets of a gene property functionally defined by a combination with another gene property. This provides more specific hypotheses for further research, and can help identify the indirect involvement of gene properties that affect a phenotype only in combination with other gene properties, for which the marginal association would be too weak to be detected in a normal GSA.

The analysis workflow is not designed as a strict protocol, as several of the analysis steps require informed decisions and interpretation of the researcher that cannot be quantified in simple thresholds.. It is therefore set up to give the researcher a reasonably wide discretion in making those decisions, without jeopardizing error rates. Selection of (marginal) associations only occurs in the first step of the workflow, with the subsequent four steps only used to provide additional information about the significant associations and giving the option of discarding some of those associations from consideration. Since no additional selection of new gene properties that were not already selected in the first step takes place in those subsequent steps, the overall error rate will be protected by the multiple testing procedure applied in the first step.

Because of this structure, the researcher is allowed some freedom to retain or discard initially significant gene properties based on substantive considerations, in cases where the statistical obtained in additional analyses is ambiguous. For example, the association of a gene property may be found to be largely but not fully explained by other more strongly associated gene properties. If the remaining unexplained association is weak there can be little confidence in any conclusions drawn from it, even if it is still below a prespecified p-value threshold. In such a scenario it may be misleading to retain it as part of the main results of the analysis as if on par with other associations, and can better be discarded from consideration and relegated to a supplementary table. But alternatively, it may be possibly to obtain additional data that can help support the association of that gene property, and such additional support would provide good grounds to still retain it as part of the results.

This analysis guideline follows the schematic of the workflow in **Figure 2** and the description in the **Methods** section. It gives additional information and recommendations for performing each of the six steps and interpreting the results. A detailed, step-by-step overview of the blood pressure analyses performed for this paper is also given in a separate section below, which can help further illustrate the process.

Step 1: initial GSA

The first step consists of a standard GSA, which is used to make the initial selection of significant gene properties to be further evaluated in the subsequent steps. Instructions for running the specific MAGMA analyses used in this workflow can be found in the MAGMA manual.

Gene annotation

A key consideration when running this initial GSA is what gene annotation to analyse. As illustrated in **Figure 1**, if a gene property is itself irrelevant to the phenotype but overlaps or is correlated with a

second gene property that is relevant, the irrelevant gene property may exhibit a significant association in the initial GSA. If the relevant gene property is also included this will most likely also have a significant association, which means that the later conditional analyses will reveal the relation between the two gene properties and will typically also correctly identify which of the two is (more likely to be) the relevant gene property.

Including larger numbers of gene properties therefore helps reduce the risk of confounding, as it increases the probability of the actually relevant gene properties are included. Moreover, even if the truly relevant gene property is not included, larger and more fine-grained annotation of genes also makes it likely that gene properties relatively similar to those relevant gene properties are included. This means that even if some gene properties are incorrectly identified, their interpretation is more likely to be closer to the actually relevant gene properties and the accuracy of the conclusions drawn is therefore still improved. An additional advantage of using more extensive annotation is that by testing more hypotheses more of them will be ruled out as probably not true (by virtue of having high p-values in a well-powered analysis), which is informative in itself.

The amount of annotation used also increases the multiple testing burden, which needs be balanced against the benefits that analysing more annotation provides. Absent a clearly specified set of a priori research questions however, it is recommended that the amount of annotation used is at least in the order of that used in the blood pressure analyses presented in this paper. If there are specific research questions relating to a more narrowly defined area of annotation however, it is important to include more general confounders in step two. This is needed to improve the reliability and generalizability of the conclusions that can be drawn.

For example, gene sets relating to the development and functioning of the heart may have been manually curated to investigate specific hypotheses about blood pressure genetics. In this case it would be important to include as possible confounders gene properties such as more general representations of the same processes and pathways, of heart expression, et cetera. If not, one runs the risk of concluding eg. that a heart-specific pathway plays a role even though that pathway is involved regardless of tissue type, or because it contains a lot of heart-expressed genes and heart expression in general is associated. Such potential confounders therefore need to be included. As they are only included as covariates and not tested themselves they do not weigh on the multiple testing burden (though nor can they be interpreted if they happen to be significant themselves).

Multiple testing correction

As in all GSA, it is recommended that a family-wise error rate (FWER) control procedure like Bonferroni correction is used rather than false discovery rate (FDR) control. For FWER control procedures control of type 1 error rates for all the individual tests (or larger subsets of tests) at an appropriate level is guaranteed as well, whereas FDR control only applies to the entire collection of tests it applies to and guarantees nothing about any individual test, or subset of tests (it also only controls the expected FDR, meaning the realised FDR can be much higher)[8]. This makes it somewhat inappropriate to interpret the results of individual tests or subsets, since those may have much higher type 1 error rates than intended. Moreover, the significance of a test when using FDR is inherently relative to which other tests were included in the control procedure, and can change considerably when that is changed (eg. a test can become significant under FDR if a set of otherwise unrelated tests of strong associations are additionally included).

When using FWER control and analysing gene properties divided over very distinct domains of annotation, an option is to use a layered correction that separately controls the FWER within each domain at an adjusted α that is itself adjusted for the number of domains. This is also the approach used in our blood pressure analyses. For example, when using Bonferroni correction the per-domain α_D would be set to the overall α divided by the number of domains D. Bonferroni correction is then applied separately for each domain using α_D rather than α, such that for each gene property in domain d the adjusted signficance threshold is $\frac{\alpha_D}{K_d} = \frac{\alpha}{D \times K_d}$ (with K_d the number of gene properties in

domain d). This approach has the advantage of making the analysis for each domain independent of the size of the other domains, which can be useful when the domain sizes are very different.

Step 2: correcting for general confounding

The aim of this step is to correct for potential confounding caused by (usually) more general factors. These will typically be relatively global variables, and are usually not included in the input annotation themselves and need to be obtained or computed separately. They are also characterised by an asymmetry relative to the gene properties of interest: if the confounder is itself associated, then the aim will be to correct the associations of all the gene properties of interest for that. This reflects an a priori presumption that any overlap in association of a gene property of interest with that of the confounder must be removed as it may bias the results for such a gene property. This in contrast to the conditional analyses in step three where overlapping associations of gene properties are treated symmetrically, and the aim of the conditional analysis is to evaluate how the two relate to each other.

Selection of the potential confounders is based on knowledge of the data, phenotype and the gene properties analysed. What are likely confounders can also vary across gene properties or groups of gene properties. Three general categories of potential general confounders that cover the majority of potential confounders can be distinguished, as described below.

The recommend criterion for deciding which gene properties to retain after this step is to apply the same significance threshold as used in step one, and discarding all gene properties that are no longer significant using that threshold when conditioned on the included confounder variables. The conditional p-value could still be very close to that threshold however, indicating that there is still a reasonable amount of association not explained by confounding for that gene property. Especially if the relative change in p-value is small this may therefore be considered too strict a criterion, and more liberal thresholds or other types of criteria could be used instead. Doing so does increase the risk of retaining gene properties for which the bulk of the association is due to known confounding however, and should be used with caution.

Data-specific confounders

These are variables that relate to the data collection and processing. These are generally technical confounders, only correlated with the observed gene association scores rather than the true associations themselves. An example is the level of coverage when using sequencing data. This may vary across genes, which can then result in different levels of noise in the data for different genes and therefore variations in the power to detect associations in those genes as well. This can then confound a gene set that happens to include a large proportion of well-covered genes, unduly inflating its observed association.

Other examples of data-specific confounders include the covariates MAGMA automatically includes in all analyses, like the number of SNPs in a gene or the level of LD (see also *Basic GSA framework* in **Methods**). For technical covariates like these, as these do not relate to the actual biology and genetics of the phenotype there is usually no interest at all in evaluating the effect they have on gene properties of interest. As such, like the automatic covariates in MAGMA, they can also be included in step 1 instead.

An example of a data-specific confounder that is more biological than technical is gene classification, such as protein-coding verses non-coding. In many analyses only protein-coding genes are included in which case it does not apply but in some cases they are, making it data (or analysis) specific. Since the protein-coding genes tend to be more strongly associated with phenotypes, it then needs to be corrected for as well.

Phenotype-specific confounders

These are variables that play a general role in the biology of the phenotype, and are likely to be related to genetic association of genes with that phenotype. A common example is tissue-specificity of genes, for example in their expression. This type of potential confounder will be relevant for most phenotypes, as almost any phenotype is likely to be specific to certain tissues or cell types to some degree. Since many other gene properties are likely to be correlated to such tissue specificity as well (eg. biological processes particular to a certain organ), variables like these have a high probability of confounding associations of gene properties in the analysis.

Often, phenotype-related variables like these will also be of interest themselves, as illustrated in the blood pressure analyses. The tissue-specific expression variables were analysed in their own right, yielding a number of significantly associated tissues that were retained. For the other domains of annotation these tissues were then included as potential confounders, as the tissue expression effects were deemed to be a much more general than the gene sets in the other domains and needed to be corrected for. In this scenario the analysis can be completed up to step four for some domains first, with some or all of the retained gene properties from that domain then included as covariates in the analyses for the other domains.

Domain-specific confounders

Sometimes potential confounders are specific to an annotation domain, reflecting properties particular to that type of annotation, and in some cases it may even be specific to a single gene property. Two examples of domain-specific confounding were used in the blood pressure analyses. The first is general miRNA target status, comprised of a gene set containing known to be a target of at least one miRNA family. This was included in the analysis of the miRNA target sets, to correct for the fact that the known miRNA target genes were on average more strongly associated with the phenotypes than other genes.

The other example is overall gene expression, which was used in the tissue expression analyses. More strongly expressed genes were more strongly associated with the phenotypes as well, which was found to be the main driver of the majority of tissue-specific expression associations. Although the overall gene expression was also included in the analyses of the other domains, this was because those domains were being corrected for the tissue-specific expression effects.

Step 3: overlapping associations

The aim of the third step is to disentangle overlapping associations of the significant gene properties retained after step two. Conditioning the different gene properties on each other can shed light on the relations between their associations, and can help in deciding which associations reflect distinct signals, which are adequately explained away by a confounding effect of another gene property, and which associations are too strongly overlapping to be distinguished from each other. Note that in all the analyses in this step, the relevant general confounders from step two should be included as covariates.

Performing the conditional analyses

The best strategy for performing this step depends on the number of significant and retained gene properties that need to be evaluated. If this is only a small number it is feasible to perform analyses for all possible pairs of gene properties. This will usually provide a clear view of the network of relations between the associations of those gene properties, some of which can then be discarded. Triples of gene properties can then be analysed together, though often the pairwise analyses are sufficiently clear and further analysis is not required.

In the more general case such a strategy would be impractical, and an alternative is needed. The degree to which gene properties can affect each other's association is roughly proportional to their own, which can be used to make the analysis more manageable. As such a stepwise procedure

can be employed, progressively analysing only the most strongly associated gene properties to map out how their associations relate to each other. For some of these a decision can then be made to discard or retain them. This is then repeated in the next step of the procedure, conditioning on all the gene properties that have already been retained and analysing the gene properties that now have the strongest conditional associations. This is continued until for each gene property a decision to discard or retain it has been made.

Although superficially similar to a typical forward selection, it is very different in practice. Forward selection usually proceeds by selecting the variable with the strongest association, conditioning the remaining variables on all previously selected variables, and repeating until no variables remain with conditional p-values below a prespecified threshold. A problem that arises with such an approach is that if two variables are strongly correlated, it is likely that only one of the two is selected. This will be the one which has the strongest association of the two, even if the difference in association is very small. This can make the selection very arbitrary, since such variables clearly reflect just a single association but only the one that happens to have the lowest p-value (which can easily change from sample to sample due to sampling variation) is selected. The aim of the stepwise approach described here is to prevent this, and instead to select such variables jointly as a pair reflecting that shared association.

An additional strategy for making this analysis step more manageable if there are many significant gene properties retained after step two is to disentangle overlapping associations within each annotation domain first, and analysing cross-domain overlap of associations only for those gene properties that are still retained at that stage. Even if no separate annotation domains were used, this approach is also possible if there functionally very distinct clusters among the gene properties to be analysed.

The motivation for such a layered approach is that within a domain or function cluster the gene properties will tend to be more similar in their function, will tend to have greater overlap and higher correlations, and will simply be smaller in number. This makes it more straightforward to reduce them to a core set of associations and discard the rest. The subsequent analyses across these domains or clusters will then also be simplified, because the number of gene properties is now already reduced and because the degree of overlap or correlation between them lower.

Conditional associations

At the core of the conditional analyses is the evaluation of the relations between the associations of pairs (or larger sets) of gene properties. In some cases this will be straightforward. If the associations of gene properties conditioned on each other are essentially the same as their marginal associations, they are independent of each other. If the association is completely removed for one of the gene properties but is still clearly present for the other, then the association of the first gene property is entirely explained by that of the second, and the first gene property can be discarded. Note that in the latter case the conditional association of the second gene property will still be reduced compared to its marginal association, possibly very substantially so. This is inherent to any conditional analysis, and as such the primary metric of interest is the relative degree of association that remains for each gene property rather than how much it was reduced.

In many cases the results of the analyses will be less clear-cut however, and making a decision can be more difficult. The table below provides an overview of the different general patterns of results for a pair of gene properties conditioned on each other (as the analyses are symmetrical, it is only given for the scenarios where the remaining association for gene property two is equal or greater than for gene property one).

Remaining association<br>Property one	 Property two	 Likely explanation
Little - none	*Little - none*	Both properties are tapping into a single underlying signal. The two properties cannot be distinguished, and should be selected and interpreted jointly, as a pair. This scenario usually occurs only when the overlap or correlation between the gene properties themselves is very pronounced.
Little - none<br>*Little - none*	*Partial*<br>*Most - all*	Gene property two is either the main driver of the shared association, or is at least closer to it. The first gene property should be discarded, selecting the second.
Partial	*Partial*	Both properties are tapping into a shared underlying signal, but this only explains part of their associations. It may be that both gene properties are relevant, but it is also possible that they are capturing an association of a third gene property not in the analysis. Depending on the relative reduction of each association either both can be selected as a pair (if similar); or the gene property with the least reduction or largest remaining association can be selected, and the other left to be selected or discarded in a later step.
Partial	*Most - all*	Gene property two cannot fully account for the association of gene property one. The second gene property should be selected, leaving the first to be selected or discarded in a later step.
Most - all	*Most - all*	The two gene properties are essentially independent, and can be selected in any order.

What constitutes 'no remaining association' is up to the researcher, but the recommended default option is nominal significance, therefore continuing the stepwise procedure until no gene properties with a nominally significant conditional association remain (not counting any already retained). In some cases a stricter criterion may be preferred however. For example, if after a number of gene properties have been retained the lowest conditional p-values are only just nominally significant, the researcher could decide there is just too little association left to allow for any useful interpretation. This would apply particularly if the original marginal association was very strong, and the proportion of it that has already been accounted for at this point therefore very high.

Step 4: detecting hidden confounding and outliers

This fourth step is specific to gene sets, and is intended to spot signs of as yet undiscovered confounding as well as outlier problems. It is based on creating QQ-plots for the genes in each set, plotting the residual Z-scores of those genes against their expected values. The latter are based on all of the genes in the data, using the sample quantiles of the residual Z-scores. These residual Z-scores are obtained from the null model for that gene set, containing all the covariates that are conditioned on but not the gene set itself.

Set-specific QQ-plots

Deviation from the plot diagonal in a QQ-plot is caused by the distribution of the observed values being different from the expected distribution. If a gene set is not associated at all its residual Z-score distribution will match that of the data as a whole. Its QQ-plot will therefore more or less follow the plot diagonal, subject to some random variation. If on the other hand a gene set is associated in its entirety for a phenotype the distribution of its residual Z-scores will differ from that of the data as a whole for every gene in the set, and the QQ-plot will therefore start to deviate from the diagonal from the first gene onward. Since stronger gene associations always mean higher Z-scores, that deviation will be upwards.

The logic behind inspecting these QQ-plots is that in the case of confounding in the superset, partial overlap and interaction scenarios (**Figure 1**), the gene set will not actually be associated in its entirety; only the genes shared with the confounding gene set or interaction term will be (if the analysed gene set is a subset of the confounder set, it cannot be detected via this method). Therefore, the Z-score distribution will be different only for that subset of genes, and only those

genes will push to deviate from the plot diagonal. The other genes have the same Z-score distribution as the data as a whole, and will therefore create an opposing pull towards the diagonal. Inherently these remaining genes will tend to have lower Z-scores. This mixture of distributions will result in a QQ-plot that, starting from the plot origin, will stay close to the diagonal initially and deviate upwards later. The relative size of the overlap with the confounding set will determine the deflection point, with smaller overlap resulting in a later (and often sharper) deflection.

QQ-plots are subject to sampling variation, and to aid their interpretation it can therefore be useful to add a confidence band to it. This will reflect the degree to which the Z-score for each gene is likely to deviate from its expected value. In practice only large upward deviations are of interest, therefore the output provided by MAGMA are for one-sided (upward) 95% confidence bands. As an additional aid to interpretation, it is also recommended that genes outside the region demarcated by the confidence band are colored differently, as it can often be difficult to spot otherwise since individual points will often blend together. For the same reason, it is recommended that some of the quantiles (for example the 25th, 50th and 75th) are separately marked. A further useful metric to compute is the proportion of genes outside the confidence region, as this can again be difficult to accurately estimate from the plot.

Interpreting the plots

The interpretation of the plots is not exact, since in practice there are different ways in which a QQ-plot can differ from the perfect exemplar of a consistent, strong, and early upward deviation. It is therefore not primarily intended to directly discard gene properties based on it, unless the issues spotted in the plot are very severe. Its main use is to inform what degree of confidence one should have in the interpretation of the association.

The primary marker to look for in the plot is the deflection point from the diagonal, and about how far in quantiles it is from the plot origin (having some of the quantiles marked clearly is therefore helpful). Differences in plot axes and in the size of gene sets means that the same degree of deviation can look quite different for different gene sets, but the confidence band can be used as a reference and having genes outside the confidence region clearly marked will further emphasize strong deviation. The computed proportion of genes outside the confidence region serves as a further clue, since this will tend to be lower when there is confounding.

This metric does need to be interpreted with caution however, as the confidence band only denotes the likely range of chance deviation per individual gene. An unconfounded gene set where all genes deviate upward but stay below the confidence band would still have a very strong association, as the chance of all genes deviating that much simultaneously is very small. This proportion therefore only serves as an aid to interpreting the plot, and should not be interpreted on its own.

Other unusual patterns can be observed in the plots as well, many of which are illustrated by the flagged QQ-plots from the blood pressure analyses shown in **Supplementary Figure 7** and **8**. A special case is the outlier pattern, where the deflection is very late and only encompasses a very small number of genes. This will tend to occur only for small gene sets, as for such sets the influence an individual gene can have is greater. If the association is driven by only a few genes then it will not have any meaningful biological interpretation, nor is there a confounding variable of potential interest that might be identified. In this scenario there is therefore little option except to discard the gene set altogether.

Interaction analysis

The properties of interaction analysis are first discussed in this general section, with issues specific to the post-hoc and exploratory analyses in steps five and six discussed in the corresponding sections below.

Interactions between gene properties

An interaction occurs between gene properties when the combination of those gene properties shows a different level of association than would have been expected based on their main effects. For example, an interaction between two gene sets occurs if genes that are in the overlap between the two sets have strongly elevated genetic associations, whereas genes present in only one of the two sets have genetic associations no different than those of genes in neither set. In this case, it is that combination of the two gene sets that makes the genes relevant to the phenotype.

Interactions can also occur in the presence of additional main effects, where for example genes in only the first gene set do have elevated genetic associations, but not nearly as much so as for genes in the overlap. Interactions can be negative as well, which would correspond to a scenario where it is not the combination of two gene properties that is relevant to a phenotype, but rather the combination of the presence of one gene set with the absence of the second gene set.

It should also be noted that if both gene sets have a main effect, genetic associations would already be expected to be higher in their overlap even if no interaction is present. In this case, a gene being in one gene set (independent of the other set) corresponds to it having an elevated genetic association; therefore, a gene being in both gene sets would be expected to have an even more strongly elevated genetic association (equal to the sum of the elevations found for the two sets).

Interactions involving continuous gene properties can be somewhat more difficult to interpret, but are conceptually no different than those between gene sets. An approximate interpretation is obtained by simply pretending they are gene sets, with a high value on the gene property corresponding to gene-set membership, and interpreting their associations as above.

In addition, interaction effects between gene properties will induce marginal associations in those gene properties, which may be detected when they are analysed in isolation. Interaction effects therefore inherently confound the marginal associations of the corresponding gene properties (see also **Figure 1D**). The reverse is also possible, with main effects of gene properties effectively acting as confounders for interaction terms. This is normally not an issue since interactions are always analysed in the interaction model, thus conditioning on those main effects. However, some gene sets are implicitly interaction terms due to the way they are defined. For example, gene sets may be created by taking general biological process sets and filtering them on being differentially expressed in the heart. This makes them interactions between the original sets and differential heart expression, and therefore need to be analysed as such to avoid false conclusions.

Note that although it is in principle possible to analyse interaction between two continuous gene properties as well as interactions between more than two gene properties, this is at present not directly supported by MAGMA. This is because such interactions are much more vulnerable to statistical artefacts and are also much more difficult to interpret even when robust. Support for such more general interactions will be added in a later version. They can however be analysed in the current version by manually creating the required interaction terms (as detailed in the MAGMA manual), though great care should be taken with interpreting the results.

Gene set by gene set interactions

One issue that is particular to gene set pairs is that to perform an interaction analysis their interaction needs to be meaningfully defined. The interaction term for two gene sets is itself a gene set, containing all the genes that overlap between the two. For many pairs of gene sets there simply is no overlap however, which means there is nothing to analyse. If there is some overlap but it is very small, the interaction is defined but is still very difficult to meaningfully interpret. If the two sets only have a few genes in common, it is hard to be sure whether this actually reflects relevant biology rather than simply chance overlap. It is therefore necessary to ensure that there is a sufficient amount of overlap, both in the absolute number of genes as in the number of genes relative to the size of either gene set.

In addition, the overlap between the gene sets should also not be too large, for essentially the same reasons. If one of the gene sets is fully contained by the other, the interaction between

them is identical to that first set and the model cannot be analysed as it is statistically singular. If the overlap is very strong but not quite complete this reduces to strong collinearity between the interaction and the gene set, which can be very unstable and as with the very small overlap is unlikely to actually reflect relevant biology.

Both the size of the overlap between the sets as well as of the partition unique to either set should therefore be sufficiently large in order for the interaction analysis to be interpretable. The thresholds used are up to the researcher, but we recommend that the overlapping and unique partitions should each contain at least 20 genes; and should also contain at least 10% of the genes in the corresponding gene set for the unique partitions and at least 10% of each gene set for the overlap. Higher values can be used as well however, and this filtering can also be used to keep the number of interactions that need to be analysed at a manageable level. This can be particularly important for an exploratory interaction analysis, where the number of interactions can quickly become prohibitively large if many gene sets are available.

Continuous gene property by gene set interactions

If one of the gene properties is continuous the interaction term is in principle always meaningfully defined, but it can be sensitive to local outliers. The interaction term between a gene set and continuous gene property is a variable that is equal to the continuous gene property for genes in the set and zero otherwise. If for a gene the value on this interaction term is very high or low relative to the other genes in the set, and if that gene also has a very high or low Z-score, it can exert a very strong influence on the interaction association. Such genes act as local outliers inside the gene set and can strongly bias the interaction.

A number of measures can be taken to prevent or detect this problem. To begin with, the interaction analysis can be restricted to only interactions with gene sets of at least a certain size, as smaller gene sets are especially vulnerable. Moreover, for smaller gene sets the interactions can also be more difficult to interpret, so they are of less interest in general. Filtering out smaller gene sets has the advantage of reducing the number of interactions that need to be tested as well, reducing multiple testing burden. We recommend 50 genes as the absolute minimum, but suggest using a cut-off of at least 100 genes unless this would too drastically limit the number of interactions that can be tested.

Even for larger sets the analysis is not immune to outlier problems, and post-hoc checks need to be performed for all significant interactions. We suggest two complementary approaches for doing so. Firstly, the analysis can be repeated with probable outliers removed from the gene set, to determine how much this would impact the interaction strength. MAGMA can provide output relevant output of Z-scores and gene property values for genes in the corresponding gene set. This can be normalized and used to make a scatter plot for that gene set, from which probable outliers can be identified. If the number of significant interactions is small, visual inspection of the scatter plots may be sufficient by itself. Otherwise, more automated ways of outlier detection can be used as well, such as described in the **Methods** for the blood pressure analyses. It is recommended that all scatter plots are inspected as well however, to ensure the outlier detection is sensible. With the likely outlier genes identified, the interaction analysis can then be rerun with those genes dropped from the gene set.

The second approach is to partition the gene set based on the continuous gene property, creating a subset of for example the top 25% or top 50% of genes (or the bottom 25% or 50% if the interaction is negative). This dichotomizes the interaction term and is therefore insensitive to high gene property values. The subset can be analysed in place of the original interaction, testing it conditional on the whole gene set and the continuous gene property. Aside from serving as an outlier check the results of this analysis can also be of interest in themselves, as the dichotomized interaction can be easier to interpret.

The results from these two post-hoc analyses can be used in different ways in deciding whether to discard an interaction. We recommend applying the same significant threshold as was used for the original interaction p-value to both, and discarding all interactions for which neither of

the two post-hoc tests is significant at this level. It can also be required that both are significant, but this may be too strict in practice. The determination of what constitutes an outlier is somewhat arbitrary, and may remove too many in some cases and lead to a weak interaction association. The partitioning is similarly subject to an arbitrary cut-off, and dichotomization removes a lot of information. The dichotomized interaction association can therefore be relatively weak as a result of this, even if there are no strong outlier effects present.

Performing interaction analysis

When performing an interaction analysis, it is recommended that the main effect of the gene properties obtained from the interaction model are compared to both their associations conditional on the other gene property as well as the marginal associations. This provides insight into the extent to which the marginal association of each gene property is accounted for by the effect of the other gene property and the interaction between them. This is mostly of interest for gene sets however, and less so for continuous gene properties. Because gene sets are binary and comparatively small compared to the total number of genes in the data, they are often inherently unable to account for much of the association of a continuous gene property.

Many of the same considerations that apply to marginal associations apply to interaction associations as well. The first is that they can be similarly subject to general confounding, and relevant confounders that have been identified for either gene property in the interaction should therefore be included as covariates. Moreover, it may be useful and sometimes necessary to include interactions between a confounder variable and one of the gene properties as well. For example, for each of the tissue-specific expression by gene set interaction analyses for the blood pressure results, an additional interaction between the gene set and overall gene expression was included. This was necessary to ensure that any interactions found would be tissue specific, rather than due to a more general interaction of that gene set with overall expression.

Secondly, interactions can overlap with and be confounded by other interactions. In the same way as in step three, it is therefore necessary to analyse the significant interactions conditional on each other, to evaluate how those interaction associations relate to each other. Moreover, interaction associations can similarly overlap with and be confounded by marginal associations, and vice versa. Conditional analyses should therefore be used to determine to which interaction and marginal associations reflect shared signals, or are confounded by each other. For example, it is possible that an interaction term is strongly associated because it overlaps strongly with a marginally associated gene set that is actually relevant to the phenotype. The reverse can also occur, with a marginal association being explained by its overlap with an interaction between two other gene sets.

When conditioning on an interaction, up to three covariates are included: the two main effects of the gene properties involved (unless already conditioned on) and the interaction term. If an association changes when conditioning on the entire interaction, this can therefore be due to any combination of main effects and interaction. In some cases it may therefore be informative to run the conditional analysis in stages to determine how much is due to the three separate components, including the main effects as covariates first, then adding the interaction term as well.

Step 5: post-hoc interaction analysis

The post-hoc interaction analyses are used to refine the significant associations retained in the previous steps. These analyses can show if those associations are confounded by an interaction, with the involvement of the gene property partially or entirely restricted to a combination with another gene property. In the process, it can also discover the indirect involvement of gene properties that were not detected before, but are interacting with the significant gene properties.

For each of the primary (ie. significant and retained from previous steps) gene properties, interactions are tested with all other gene properties for which the interaction is meaningfully defined (as defined by the size and overlap criteria outlined above). As these are post-hoc analyses

multiple testing correction is applied separately for each primary gene property, correcting for the number of interactions tested for that gene property.

For interactions between gene sets, it is recommended that only one-sided tests for positive interactions are performed, to improve power. Although it is possible for there to be negative interactions as well, since the primary gene property has a significant marginal association these are much less likely than positive interactions, as a negative gene-set pair interaction will weaken its marginal association. For continuous gene properties, the testing direction should typically be the same as was used in the previous steps for that gene property.

Step 6: exploratory interaction analysis

The purpose of the exploratory interaction analysis is to uncover additional associations that may have been missed in the previous five steps. Some interactions may have had strong enough marginal associations to be significant in the initial GSA, or because gene properties involved also had main effects. For many interactions this will not be the case however, especially negative interactions, and detecting these interactions will require testing them directly. This sixth step is designated as optional because it is independent of the previous steps, but as it could provide valuable additional insight into the phenotype that is otherwise likely to be missed, running it is recommended.

To select which interactions to analyse, it is recommended that an initial subset of gene properties is selected first, and to then run all interactions of those gene properties with all other gene properties (including others in the initial subset) for which the interaction is meaningfully defined (see above). Unless the number of available gene properties is relatively small, running all possible interactions is likely to result in a prohibitively high multiple testing burden, and some filtering is therefore usually needed.

The most straightforward way of generating the initial subset is by selecting based on marginal associations. For almost any interaction, it is likely that at least one of the gene properties involved will show some weak marginal association as well, which can therefore be used to narrow down the number tested interactions somewhat without an undue risk of filtering out any that are actually associated. As long as such a marginal association criterion is imposed on only one of the gene properties in an interaction pair, family-wise type 1 error rates are still correctly maintained. For generating the initial subset a liberal criterion like FDR-corrected significance or even just nominal significance can be used, also depending on how many interactions to be tested this would yield.

When running the exploratory interaction analysis, it is also recommended that two-sided tests are used. This allows negative interactions to be detected as well, which are much more likely to be present here than they were in step five. Since this is an independent analysis, family-wise error rate control should be applied for the total number of interactions tested.

DETAILED OVERVIEW OF BLOOD PRESSURE ANALYSIS

This section contains a detailed description of all the analysis steps as applied to the analysis of the blood pressure phenotypes. Note that the three phenotypes were analysed independently of each other, with multiple testing corrections applied separately to each phenotype.

Step 1: initial GSA

The main GSA was performed with MAGMA 1.07, using the settings described in the Methods section. Analysis was performed separately for each of the three phenotypes, with multiple testing correction applied per phenotype. Bonferroni correction was used with an overall α of 0.05. A correction was first applied for the number of domains (five in total), setting the per-domain α to $0.05/5 = 0.01$. Bonferroni correction was then applied within each domain at this per-domain α.

This procedure was used to ensure that the overall family-wise error rate is controlled at the intended 5% while preventing the Gene Ontology - Biological Process domain (which contains 72% of all the gene properties analysed) from dominating the other four domains. It should be noted that the Biological Process domain yielded by far the most significant gene sets, despite the comparatively less strict threshold in the other three gene set domains.

A summary of the results from this initial GSA can be found in **Table 1**, with all the individual results available in **Supplementary Tables 9** through **13** (in the **Supplementary Data**). Details on the significant results are also presented in **Table 2** for gene properties retained at the end of the extended analysis workflow, and **Supplementary Tables 1** and **2** for gene properties which were discarded.

Step 2: correcting for general confounding

A number of likely domain- and phenotype-specific confounders were selected to correct for possible confounding of the initial results. These are in addition to the data covariates already automatically included by MAGMA in the initial analysis (number of SNPs, estimated level of LD and inverse mean MAC per gene; plus the log transformation of these three variables). They are conditioned on in all subsequent analyses as well, unless explicitly noted otherwise. The p-values for all individual gene properties before and after correcting for the confounders can be found in **Supplementary Tables 9** through **13** (in the **Supplementary Data**). Only initially significant gene properties which remained significant after conditioning on the potential confounders at the same significance threshold as used in step one were retained for further analysis. Unless otherwise specified, p-values for marginal associations reported in the paper are from the analysis in this step.

For the tissue expression domain the overall gene expression level was conditioned on, computed as the mean expression across tissues for each gene. This was found to be very strongly associated for all three blood pressure phenotypes (**Supplementary Table 5**), with more strongly expressed genes also on average exhibiting stronger genetic associations with the phenotypes. As shown in **Table 1** this overall expression acts as a very strong confounder of the individual tissue expression levels, with the number of significant associations dropping from 145 to 17.

For the miRNA target sets a domain-specific confounding variable was conditioned on as well, reflecting a global effect for (known) miRNA targets. This confounder was strongly associated for SBP and PP (**Supplementary Table 5**), with genes known to be a target of at least one miRNA family on average more strongly associated with the phenotypes than other genes. Conditioning on this removed two of the three significant associations (all for PP).

In addition to these domain-specific confounders, for all four gene-set domains a number of gene expression variables were included as phenotype-specific confounders as well. From the tissue

expression analysis it is clear that genetic association with blood pressure phenotypes is strongly associated with tissue-specific gene expression. As these represent effects that are much more global than those of any of the gene sets, they were included as general confounders here. The tissues used were coronary and tibial artery, heart (atrial appendage) and uterus. The overall gene expression was included as a covariate as well. These tissues were chosen based on the tissue expression results from step 3. Although not all these tissues were significant for all three phenotypes, the same tissues were included for each to simplify comparison of results across the phenotypes. All tissues that were significant for at least two of the phenotypes were used, as well as the heart (atrial appendage) expression as this represented a third distinct signal for PP.

The selected variables were found to indeed act as confounders for many of the significant gene sets, as shown in **Table 1**. The last remaining significant miRNA target set was discarded, and for the Gene Ontology domains only 35 of the 71 significant gene sets were retained. It should be noted that for many of the gene sets the confounding appears only partial, with p-values exceeding the significance threshold but still relatively low (see **Supplementary Figure 3**). These gene sets therefore may still contain some relevant association, though as the same figure shows much of that association is subsequently accounted for by the gene sets that were retained at the end of the analysis workflow.

Step 3: overlapping associations

The significant and retained gene properties within each domain were conditioned on each other, to evaluate the relation between their associations as well as discard any gene properties for which the associations were adequately explained by other gene properties. A forward selection procedure was used to do so, as described in the analysis guideline above. In each step of this procedure, gene properties were conditioned on those gene properties already selected in previous selection steps (and general confounders), and the next gene property was selected based on their conditional p-values.

In any step, if multiple gene properties were more or less tied for the lowest conditional p-value, conditional analyses were performed conditioning these gene properties on each other (and all other variables already conditioning on) to determine the relation between their associations. If any of these gene properties were found to strongly and more or less symmetrically overlap (indicating that they likely tap into the same, shared association), their relation was noted and they were selected jointly. This procedure continued until no gene properties with conditional p-values below 0.05 (uncorrected) remained. The remaining gene properties were then discarded.

In some cases this full stepwise approach was required due to the larger number of gene properties that needed to be analysed. In other cases the analysis could be simplified because this number was smaller, and it was therefore feasible to perform analysis for all possible pairwise combinations. The details for all these analyses are given below

Tissue-specific expression

Conditioning the three tissues for DBP on each other revealed a great deal of overlap in their associations, as shown in this table:

	**P-value conditional on**			
**Tissue**	**None**	**AC**	**AT**	**UT**
Artery (coronary) (AC)	1.13e-4	-	0.152	0.0262
Artery (tibial) (AT)	8.83e-5	0.301	-	0.0311
Uterus (UT)	4.78e-5	0.0148	0.00967	-

The two artery associations fully explained each other, and clearly represent only a single, shared signal. This strongly overlapped with the uterus expression association as well, but not entirely. As such, being most strongly associated the uterus association was selected first, followed by the two artery tissues selected as a pair. Although the number of tissues was therefore not reduced, this analysis shows that there are likely two distinct signals driving their associations.

For the PP results there was also a pronounced overlap in associations. Of the fourteen associated tissues seven could be discarded, with the remaining seven corresponding to three distinct but correlated signals centered on arterial expression, heart expression and expression in a cluster of female reproductive organs. Strongest by far were the associations for the three artery tissues, which were too strongly correlated with each other to distinguish and were therefore selected as a group.

Conditioning on the arterial expression removed much of the association in the other tissues though not all, with the heart (atrial appendage) the most distinct of the remainder (see figure below; red lines denote nominal and Bonferroni-corrected significance thresholds). Conditioning on the heart expression as well left a weakened but still distinct signal in the trio of endocervix, ovary and uterus. Of these, the uterus expression had the strongest initial association and was also the only one associated for the other phenotypes, but after conditioning on artery and heart expression the three associations could no longer be distinguished and were again selected as a group.

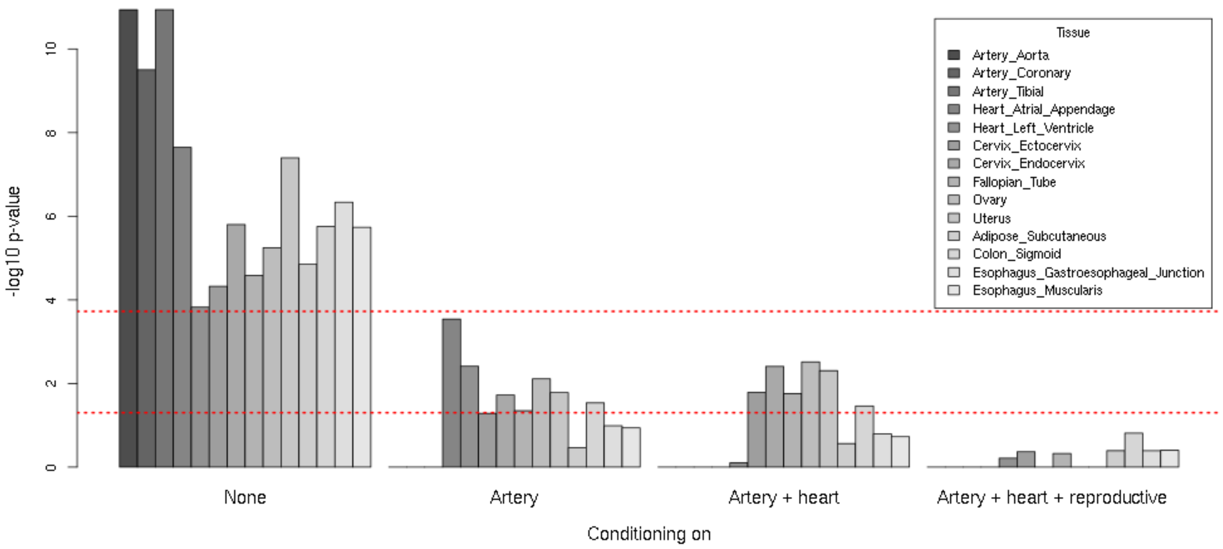

Gene Ontology gene sets

For the Biological Process domain, conditional analysis was required for all three phenotypes. For SBP, the results for the six gene sets were straightforward. There was strong overlap in the associations of *cardiocyte differentiation* (95 genes) and its subset *cardiac muscle cell differentiation* (73 genes). Of these the *cardiocyte differentiation* set had the stronger marginal association and could fully account for the association in its subset, which had a conditional p-value of 0.666 compared. With a conditional p-value of 0.00166 remaining for its parent despite sharing the bulk of its genes, this meant that *cardiac muscle cell differentiation* could be discarded. There was no further overlap of note in the associations of the remaining five gene sets, and they were therefore all retained.

The six gene sets for DBP were similarly straightforward to analyse. The *nitric oxide metabolic process* (15 genes) and *reactive oxygen species biosynthetic process* (21 genes) strongly overlapped both in genes, with 13 genes in common; and in association, with conditional p-values of 0.0201 and 0.0101 respectively. They were therefore selected as a pair, as they clearly reflect only a single

shared association. Similarly, *regulation of urine volume* (19 genes) and its subset *positive regulation of urine volume* (14 genes) share a single association. The evidence slightly favours the latter with a conditional p-value of 0.0398, against 0.187 for its parent. Although the difference is far from decisive, given the strong similarity in the biological processes these two sets correspond to the *regulation of urine volume* was discarded, retaining *positive regulation of urine volume*.

With 16 significant and retained gene sets, the analysis for PP was more complicated, and the stepwise approach had to be used. The p-values reported below are from the stepwise analysis, and are therefore for associations also conditioned on all already retained gene sets.

The strongest association was found for the *cardiovascular system development* and *circulatory system development*; as these are fully identical, analyses were only performed for *cardiovascular system development*. This association fully explained the association of *heart development* (their subset; conditional p-value of 0.402) and *anatomical structure formation involved in morphogenesis* (overlapping with about 50% of its genes; conditional p-value of 0.0230, but later pushed past the nominal significance threshold as well), which were both discarded; conditional p-values for *cardiovascular system development* conditioned on these sets were 0.000154 and 3.74e-5 respectively.

Using the forward selection, several more sets could straightforwardly be selected, with no ambiguity in their relation to other gene sets: the *negative regulation of cellular senescence* set, explaining all association from its parent set *negative regulation of cell aging* (respective conditional p-values of 0.00328 and 0.343); the *negative regulation of smooth muscle cell proliferation* set (36 genes) and its parent set *regulation of smooth muscle cell proliferation* (98 genes), which shared a single association (conditional p-values of 0.0111 and 0.0118) and were selected as a pair; and the *cell proliferation* set, not very strongly overlapping with any of the already selected or remaining gene sets.

After this, there was a cluster of three related gene sets still showing association: *cardiocyte differentiation* (p-value of 5.014e-5), *cardiac muscle cell differentiation* (p-value of 0.000186) and *cardiac muscle tissue development* (p-value of 0.00543). The *cardiac muscle tissue development* was fully accounted for by either of the other two sets (conditional p-values of 0.466 and 0.453) without strongly affect those sets in return (conditional p-values of 0.00167 and 0.00655 respectively), and could therefore be discarded. For the first two sets, conditional p-values were 0.0565 and 0.382 when conditioning on the other, giving the edge to *cardiocyte differentiation*. The *cardiac muscle cell differentiation* set was therefore discarded.

The *mesenchyme development* set was then selected at the expense of *mesenchymal cell differentiation*, based on respective conditional p-values of 0.0172 (down from 0.000337) and 0.615 (down from 0.00381). After this only *regulation of transcription from RNA polymerase II promotor* remained, with a conditional p-value of 0.00763. Although markedly reduced compared to its marginal p-value of 9.97e-7, it still showed some remaining and unexplained association and was therefore retained as well.

For the other two Gene Ontology domains, for SBP and DBP at most one gene set was retained after step 2 per domain, and conditional analysis was not needed. For PP there were two gene sets remaining for both domains. For Cellular Component these were *cytoskeleton* (1882 genes) and its subset *actin cytoskeleton* (430 genes). Conditioning on each other partially accounted for both associations, dropping from 1.88e-7 to 0.000470 and from 1.08e-6 to 0.00296 respectively. Since the sets are nested and the larger *cytoskeleton* set has the stronger marginal and conditional association, this suggests the possibility that there is a third gene set intermediate between these two that drives the observed associations in both. The two sets are retained, but as a pair. Conditional analysis for the two Molecular Function PP-associated gene sets shows that the two are effectively independent of each other.

Further analyses were also performed to determine if there was overlap in associations across the Gene Ontology domains. Each of the significant and retained gene sets was conditioned on all the retained gene sets from the other two domains simultaneously. There were no large

changes in p-values for any of these gene sets however, indicating that the associations in the different domains are independent.

Step 4: detecting hidden confounding and outliers

Set-specific QQ-plots were created for all the significant and retained gene sets, plotting the observed residual gene Z-scores against their expected values, with the latter based on the corresponding quantiles of residual Z-scores for all of the genes in the data. An asymmetrical 95% confidence band was added to provide an indication of how much deviation would be expected by chance, and the proportion of genes for which the observed residual Z-score fell outside the confidence band was computed as an additional summary metric.

Each of the plots was inspected for signs of hidden confounding or outlier problems. In total, six gene sets were flagged as potentially problematic, with a seventh being discarded altogether. It is of note that these included six of the seven remaining cellular component and molecular function gene sets, compared to one out of nineteen for the biological process domain. The QQ-plots for these sets are all shown in **Supplementary Figure 7** and **8**, with an eighth plot for the unflagged *cardiovascular system development* set to serve as an additional comparison.

The six gene sets flagged in this step are still retained and are part of the final results, but more caution may be needed when interpreting their associations. This does not necessarily apply to all gene sets flagged this way, as not all unusual-looking are indicative of real problems. This applies for example to the *peptide hormone binding* set (**Supplementary Figure 8C**), which was flagged because only 20% of genes in this set exceeds the confidence band. Except for one gene they are very consistently elevated and otherwise very close to the confidence band, so on balance there is likely no problem with this association. For other gene sets there is reason to worry however, with the plots giving reason to suspect at least an increased likelihood that the association is the result of confounding due to some unknown other gene property, or otherwise compromised.

Interpretation of individual plots

From the biological process domain, only the *negative regulation of transcription from RNA polymerase II promotor* set associated for SBP was flagged. Although with almost 88% of genes outside the confidence band there appears to be a consistent signal in the gene set, a point of concern is the lower tail. There is a subset of genes there which is comfortably inside the confidence band and in fact partially and fairly markedly dips below the plot diagonal. Given the otherwise strong elevation, one possibility is that the actual involvement with SBP is due to a more specific subprocess of this gene set, though in that case it would still have a similar interpretation. The plot for this gene set is therefore not deeply problematic, but it does need to be taken into consideration.

A similar pattern is observed for the cellular components *actin cytoskeleton* and its parent *cytoskeleton*, both associated for PP. It is stronger for these sets, especially for *cytoskeleton*, and the fact that it applies to both of these nested sets suggests that both may be overlapping with another, unknown gene property that is actually involved in PP. This overlap would have to be quite strong so it might also be relatively close in interpretation, but it is impossible to be sure. In contrast to *negative regulation of transcription from RNA polymerase II promotor*, involvement of a subprocess is less likely here. A confounding subprocess intermediate between the two sets is unlikely as in this case the *actin cytoskeleton* set would be a subset of this confounder and should have no issues with its plot. On the other hand, a confounding subprocess that is a subset of *actin skeleton* is also unlikely since in that case the association of *cytoskeleton* should have disappeared when conditioning on *actin cytoskeleton* in step three, which it did not do.

The third cellular component set is *T tubule* for SBP. The plot here is more unusual, with a relative depression in the middle rather than at the tail. This may indicate that the association results from this set overlapping with possibly multiple other truly associated sets. On the other hand, all genes are consistently above the plot diagonal so it may just be an unusual distribution of gene

associations for this set. As with *negative regulation of transcription from RNA polymerase II promotor*, the plot is unusual enough that it needs to be flagged, but the issues it shows do not seem grave enough to be deeply concerning.

Much more problematic is the *high-voltage gated calcium channel activity* molecular function set for SBP. Although here as well all genes are consistently above the diagonal, the top two genes of this set deviate very strongly from their expected value as well as the confidence band, whereas the majority of other genes do not. Because the set is only 10 genes large, it is probable that its association is largely driven by those two genes, which effectively constitute a pair of outliers in this set. For this reason the set was discarded from the results, since it would be impossible to rely on the veracity of its association.

Also from the molecular function domain are *cell adhesion molecule binding* and *peptide hormone binding*, both for PP. The first looks quite problematic, with a depressed tail even larger than some of the previous plots and a relatively lacklustre middle as well. On this basis, the association for *cell adhesion molecule binding* seems too unreliable to interpret, and could be discarded altogether on this bases. However, this set is relatively large (180 genes) and does show a reasonably strong association. A follow-up study bringing additional annotation related to this set or the genes it contains to bear on it may reveal a reliable underlying association that can be interpreted, and could therefore provide insight into the pulse pressure phenotype even if *cell adhesion molecule binding* is found to be entirely irrelevant.

For *peptide hormone binding* there is likely no serious problem. The plot is flagged because a large majority of genes is inside the confidence band. They are still consistently elevated above the plot diagonal however, excepting one gene, and there are no clear outliers that could be unduly inflating the association. Despite the unusual QQ-plot, it is therefore likely safe to retain and interpret this gene set.

Step 5: post-hoc interaction analysis

Tissue expression by gene set interactions

Post-hoc interaction analysis was performed for the significant tissues, analysing positive interactions with gene sets. To allow for more comparison across the phenotypes the same four tissues were used for each, which were the same tissues also selected as general confounders in step two: coronary and tibial artery, heart (atrial appendage) and uterus. In addition to the tissue-specific expression, interactions were also analysed for the overall gene expression with the gene sets. No significant interactions were found for the overall expression, but it was still included in all the tissue-specific interaction analyses (both the overall expression main effect and its interaction with the gene set being used in an analysis), to ensure tissue-specific interactions were not confounded by non-specific interactions that were too weak to reach significance themselves.

Since the interaction analysis between a gene set and a continuous variable can be very vulnerable to outliers if the set is small, only gene sets of at least 100 genes (after discarding genes with no expression values) were used. This led to 1,495 interactions being tested for each of the tissues. As these are post-hoc analyses the Bonferroni correction was applied per tissue, resulting in a total of 60 significant interactions. Outlier checks were performed for these 60 interactions as described in **Methods.** For each interaction the likely outliers were removed from the gene set and the interaction was reanalysed. An additional analysis was also performed of the subset of the top 25% tissue-expressed genes within the set, conditioning on the whole gene set and the tissue expression. If neither analysis was significant at the original threshold of 0.05/1495 = 3.34e-5, the interaction was discarded (see **Supplementary Table 3** for all discarded interactions). After these checks, 33 interactions were retained: 20 for SBP, 7 for DBP and 6 for PP.

Conditional analyses were then performed to evaluate the relations between the interactions, since there was considerable overlap in both the gene sets involved (with some gene sets interacting with multiple tissues) and the tissue expression levels were strongly correlated. This

led to seven interactions for SBP and one for DBP being discarded. There was considerable overlap among the other interactions as well, with many of the interactions therefore selected in groups reflecting shared signals (see **Table 3**). Note that these conditional analyses were performed with the likely outliers removed, so p-values reported below may differ from those in **Table 3** and **Supplementary Table 14** (in the **Supplementary Data**). When conditioning on an interaction, both the interaction term itself as well as the tissue expression and gene set it is composed of are included as covariates in the model.

For DBP the interaction between heart expression and *purine-containing compound biosynthetic process* was discarded, as its association was accounted for by the interaction of coronary artery expression and that same gene set (with the p-value going from 0.00062 to 0.087).

For SBP, there was a cluster of interaction of coronary artery expression with the three sets *positive regulation of cyclic nucleotide metabolic process*, *positive regulation of nucleotide metabolic process* and *positive regulation of purine metabolic process*, and of heart expression with *positive regulation of cyclic nucleotide metabolic process* and *regulation of cyclic nucleotide metabolic process*. These five interactions were almost entirely explained by a second cluster of interactions reflecting a single interaction signal, consisting of the interactions of both coronary and tibial artery expression with *nucleoside phosphate biosynthetic process* and *purine containing compound biosynthetic process*. Although four of the five interactions remained nominally significant after conditioning on all the selected interaction, they were very weak (all p-values were greater than 0.01). They were therefore considered to be of too little further interest, and were consequently discarded.

The interactions of heart expression with *intracellular signal transduction* and *second messenger-mediated signaling* were also discarded. The first was partly explained by the interaction of heart expression with *receptor signaling protein activity*, and for the rest by the combination of other retained interactions during the stepwise selection process. The second interaction was accounted for by the combination of the interactions of heart expression with *receptor signaling protein activity* and with *vascular process in circulatory system*.

Among the retained interactions, the shared associations (**Table 3**) were readily explained by the tissues and gene sets involved. The interactions for uterus expression were with three gene sets all related to sexual development which were almost entirely nested in each other. The *nucleoside phosphate biosynthetic process* and *purine containing compound biosynthetic process* sets, interacting with several tissues, strongly overlap: the *purine containing compound biosynthetic process* is almost entirely contained by *nucleoside phosphate biosynthetic process*.

The *vascular process in circulatory system* and *regulation of blood pressure* gene sets are of almost equal size and overlap with about 40% of their genes, explaining the overlap in their interactions with heart expression for DBP. Of note however is that although for DBP the overlap of these two associations was complete, for SBP it was not; conditional p-values for these interactions were 6.8e-5 and 0.0013 respectively (whereas for DBP these were 0.651 and 0.391). Although clearly not independent in view of the difference with their original interaction p-values, for SBP they do not appear to fully overlap either.

Gene set by gene set interactions

Interactions were tested for all significant and retained gene sets with all other gene sets that meaningfully overlapped with them (see **Methods** for details). A total of 1,153 interactions were tested (not counting duplicates for *circulatory system development*, identical to *cardiovascular system development*), of which 188 for SBP, 86 for DBP and 879 for PP. Eight of these were significant, all of which for PP. Two of those were discarded after conditional analysis, with the six retained interactions given in **Table 4**. When conditioning on an interaction, both the interaction term itself as well as the two gene sets it was composed of were included as covariates in the analysis.

Conditional analysis was necessary as multiple interactions were found involving the same gene sets. The *cardiovascular system development* set was found to interact with two nested gene

sets, *chemical homeostasis* and its parent set *homeostatic process*. The two interactions were found to reflect a single shared signal, which also explained a considerable portion of the original association of *cardiovascular system development*. The *homeostatic process* set also interacted with *regulation of transcription from RNA polymerase II promotor*, but this was found to be largely distinct from its interaction with *cardiovascular system development*.

For *cell proliferation* a total of four significant interactions were found, with four similar and overlapping gene sets. As shown in the table below, the interactions with *positive regulation of establishment of protein localization* and *positive regulation of transport* were explained by the interaction with *regulation of intracellular transport*. The latter did not account for much of the interaction with *regulation of intracellular signal transduction* however, and therefore these two were both retained. Together they also account for almost all of the marginal association of *cell proliferation* which goes from 1.6e-8 to a main effect p-value of 0.015 when conditioning on both interactions simultaneously.

		**P-value of interaction with *Cell proliferation*, conditioned on**				
**Gene set**	**No. of genes**	**None**	**PPL**	**PT**	**RIT**	**RIST**
Positive regulation of establishment of protein localization (PPL)	498	0.000186	-	0.335	0.142	0.00269
Positive regulation of transport (PT)	907	0.000135	0.0894	-	0.0971	0.00190
Regulation of intracellular transport (RIT)	596	7.62e-6	0.00463	0.00560	-	0.000204
Regulation of intracellular signal transduction (RIST)	1581	0.000204	0.00271	0.00278	0.00526	-

Step 6: exploratory interaction analysis

The exploratory interaction analysis was performed for all pairs of gene sets for which there was meaningful overlap (see also **Methods**), and for which at least one of the sets was significant after FDR correction (applied separately to each of the four gene set domains) at an α of 0.05. Exploratory interaction analysis is also possible between gene sets and continuous variables, but because of the strong correlations between the tissue-specific expression levels and their relatively small number. Given this, this would in all likelihood reveal only interactions largely or wholly overlapping with those found in step five, and therefore unnecessarily increase the multiple testing burden.

For the exploratory interaction analysis a two-sided test was performed on the interaction term, rather than only testing for positive interactions. This allows for the detection of negative interactions, which reflects a scenario where one gene set is involved in a phenotype specifically in the absence of the other gene set. In this scenario the marginal association of that gene set would be lower than the main effect in the interaction model, and possibly too small to reliably detect in a normal GSA. By testing for negative interactions, these associations can potentially be detected indirectly.

The number of interactions tested varied across the phenotypes as a result of the FDR-based inclusion criterion, with 5,652 tested for SBP, 3,213 for DBP, and 13,009 for PP. Of these interactions, none were significant after Bonferroni correction. As shown in **Supplementary Figure 4** (top row) however, there is evidence for association in these analyses even though no individual interaction was strong enough to be detected. As such, to illustrate the utility of the exploratory interaction analysis step in the workflow, an FDR correction was applied to the results instead. This yielded 7 associations for SBP and 12 for PP (see **Supplementary Table 4**). These are not intended to be interpreted in relation to blood pressure genetics, but are only presented for illustration of the method. Given that these became significant however, it is clear that somewhat stronger interactions or data where power was somewhat higher would be likely to yield significant associations under a more stringent family-wise error rate correction as well.

Note that no further conditional analyses were performed for these interactions, and given the likely overlap in the involved gene sets and the repeated occurrence of some sets in different interactions it is clear that some of these interactions reflect shared signals. Some of these interactions were already detected in the post-hoc interaction analyses, though some of them now also show up for SBP. Others do involve gene sets that were not previously implicated, and this applies in particular to the four negative interactions. Of these, three involve a gene set for which the main effect would be significant under the threshold applied for that set in the initial GSA, demonstrating the ability of this analysis to uncover associations that are quite strong but partially masked by an overlap with an irrelevant second gene property that essentially acts as dead weight.

SIMULATION STUDY

To generate the phenotypes for the simulations, a genetic component was constructed from SNPs in the 1,000 designated causal (ie. with genetic effect on the phenotype) genes. For each causal gene, 10% of the SNPs were randomly selected as effect SNPs (rounding to the nearest whole number, or to one if this would be zero). Genotypes for these SNPs were extracted from the data and normalized. Per gene, the genotypes were then summed, renormalized, and scaled by the log of the number of effect SNPs in that gene. These were then summed across the causal genes and again renormalized. Phenotypes were generated by adding normally distributed noise with a mean of zero and a variance of $\frac{1}{h^2} - 1$, for the desired explained heritability of $h^2 = 0.1$ (as this reflects only the additive heritability explained by common genic SNPs, it is set to a relatively low value). Simulated GWAS results were then generated as described in the **Methods** section. In each replicate, ten phenotypes were simulated with new random noise, and SNP p-values were meta-analysed to obtain results at the desired sample sizes of 10,000, 50,000 and 100,000.

The motivation for this setup was such that different gene-set level scenarios could efficiently be simulated without the need to generate new sets of GWAS results for each replicate of each simulation condition. Different scenarios can now be simulated by generated gene sets with different patterns of overlap and proportions of causal genes. It should be noted however that this strategy provides only approximation of the null models of the conditional and interaction GSA models, since the latter is defined in terms of the continuous gene Z-scores, rather than a binary and latent 'causal gene' status. This is shown in the results as well, where in some cases the type 1 error rates deviate somewhat more strongly (both upward and downward) from the nominal significant level than simulation noise can account for. These deviations are minor however, and the simulations can therefore still be used to reliable evaluate the conditional and interaction GSA models.

The main aim of the simulations was to verify the type 1 error rates of the models as well as to show the bias that can result from using the standard marginal association GSA. Different scenarios were simulated to do so, in each case first generating an initial gene set with an assigned effect, then creating additional gene sets overlapping with it to be tested (or for the interaction between them to be tested). Effects were assigned to a gene set by setting a proportion of the genes in the set to be causal, randomly sampling those from the designated causal genes available. Other genes were randomly sampled from the remaining genes; to remove some of the simulation noise, these were sampled in the proportion of causal to non-causal genes in those remaining genes. The effect sizes noted in the paper are therefore the percentage of genes explicitly assigned to be causal in a gene set, the actual proportion of causal genes in each set will be slightly higher.

For the conditional model simulations three general overlap scenarios were used, with the tested gene sets either a subset or superset of the initial gene set, or a set partially overlapping with it. For the subset scenario, the sizes of the initial and tested gene sets were either 100 and 50; 200 and 50; or 200 and 100. For the superset scenario, the sizes were 50 and 100; 50 and 200; or 100 and 200. For the partial overlap scenario both gene sets were 100 genes, and the overlap was set at either 25, 50 or 75 genes. For the subset and partial overlap scenarios, the overlapping subset was

created by randomly sampling the requisite number of genes from the initial gene set. The effect size of the initial gene set was either 0%, 50% or 100% causal genes.

For the interaction model simulations, gene sets were set to 200 genes, with an overlap of 50 genes. Main effects for the initial and second gene set were either 0% and 0%, 75% and 0%, or 75% and 75% (in the latter case the effect was generated for the initial set first, then was generated for the non-overlapping part of the second set). The aim of these simulations was only to evaluate the interaction term in the context of main effects. Although main effects can also be confounded by interactions, this is essentially equivalent to the subset scenario in the conditional model simulations and therefore did not require additional simulations.

Results for all the simulations can be found in **Supplementary Tables 17** and **18** (in the **Supplementary Data**). A subset of the results is also shown in **Supplementary Figures 1** and **2**, for two of the five significance thresholds. For **Supplementary Figure 1** the error rates were averaged over the different gene set size and overlap combinations, to simplify the plots.

Although simulations were only performed for gene sets and not for continuous gene properties, the model structure is the same in both cases and the results can therefore be safely extrapolated to continuous gene properties as well.

REPLICATION ANALYSIS

Data

To further validate the results of our analyses, we performed a replication in an independent sample. The data we used for this was the ICBP GWAS results data from 2011[9]. The data consists of SNP summary statistics based on an analysis of 69,395 individuals, with results for 2,650,286 SNPs for SBP, 2,673,126 SNPs for DBP, and 2,585,510 SNPs for PP.

The European panel of the 1,000 Genomes phase 3 data[3] was used as reference data for the analysis. Annotation of SNPs to genes was performed using the same gene location definitions and window settings as for the main analysis, which resulted in 17,841 autosomal protein-coding genes with at least one SNP mapped to them, and 42.4% of SNPs in the data (out of 2,584,592 SNPs present in both the 1,000 Genomes data and the ICBP results) being mapped to at least one of these genes. Note that the number of genes available for each phenotype was somewhat lower, with 17,834 genes analysed for SBP, 17,836 genes for DBP, and 17,815 for PP.

Analysis of this data was done with MAGMA 1.07 using the summary statistics option, with the same SNP-wise (Multi) gene analysis model. The burden scoring mechanism was not used as this is only available for raw genotype analysis. Replication analysis was then performed for all the associations listed in **Table 2, 3** and **4**, using the same general confounders as were used in the main analysis.

Results

We note that the ICBP data is not ideal as a replication sample. Due to the smaller sample size and considerably lower number of available SNPs, the gene associations obtained from the ICBP data are much noisier. With the difference in SNP coverage the number of genes that can be included in the analysis is also smaller, with between 460 and 481 (depending on the phenotype) UK Biobank genes having no gene association Z-scores in the ICBP data (in addition, 11 ICBP genes were not available in the UK Biobank analysis). As a result the correlations between UK Biobank and ICBP gene Z-scores were very low: 0.306 for SBP, 0.299 for DBP, and 0.209 for PP.

Overall there was also little detectable association in the ICBP data at the gene annotation level. When analysing all of the available gene properties, even when omitting the general confounders from the analysis, only a single gene property was significant (a molecular function gene set for SBP; it was not associated in the UK Biobank analysis, though it did have a moderately low p-value there). This is in stark contrast to the results from step 1 of the UK Biobank analyses, which yielded 145 significant tissue expression associations and 74 significant gene sets.

Because of the weak correlations between the two samples, the conclusions that can be drawn from the replication analysis are necessarily limited. No other publically available GWAS results for blood pressure could be found however; the more recent blood pressure GWASs we found either made no summary statistics available, or only for SNPs with p-values below a set threshold (whereas GSA requires all SNPs as input for the analysis).

Despite the limitations of the replication sample, the replication was still reasonably successful. Results for the replication analysis are given in **Supplementary Table 6, 7** and **8**. For both the conditional associations and the interactions, some of the UK Biobank findings did replicate in the ICBP sample. Moreover, the replicated analyses were generally enriched for lower p-values: of the total of 67 associations that were analysed for the replication, 61 had a p-value below 0.5 (91%) and 32 were nominally significant (48%), far more than would be expected by chance.

GENE-SET ANALYSES IN RECENT BLOOD PRESSURE GWAS

Overview

In the recent blood pressure GWAS[10-15], some GSA has been performed as a secondary analysis. We have compiled an overview of the results of those analyses here as a comparison with our own results, to see if they converge on the same conclusions. This comparison is complicated by a number of factors however.

Firstly, the gene properties used in these analyses are quite variable. In some of the GSAs Gene Ontology terms were used, or variables relating to tissue-specificity, in which case the comparison with our own results was more straightforward. A variety of other annotation such as databases of known canonical pathways were used as well however, for which there was generally no obvious mapping to any of the annotation used on the annotation used here.

Secondly, the methods used for the analyses were varied as well, and further complicate the comparison. To an extent this is unavoidable since the analysis workflow presented in this paper is designed specifically to go beyond traditional GSA, clear differences exist between the basic MAGMA GSA used in step one and the models used in other papers.

Many of the methods used (MAGENTA[16], DAVID[17], IPA[18], GeneGO) are implemented as variations on Fisher's exact or hypergeometric tests applied to a two by two table, based on the gene set and a dichotomised gene list reflecting blood pressure association status. With this dichotomisation the association of a gene with a phenotype is characterised in a very different way than the continuous association score used in MAGMA. Moreover, neither these nor the other methods (FORGE[19], DEPICT[20]) correct for likely technical confounders such as gene size that can cause a potentially severe bias[21]. For DEPICT there is a further complication, as the gene sets it analyses are reconstituted based on gene coexpression data. These reconstituted gene sets are only weakly correlated with the original gene sets they are based on, which means the results are not directly comparable.

A third complication is that many of the GSAs are based on genetic associations collapsed over different blood pressure phenotypes, and which blood pressure phenotypes were used also varied from SBP and DBP only, to using SBP, DBP, PP, mean arterial pressure (MAP) and hypertension (HT). As the results in our analyses show the different phenotypes appear to have distinctly different functional architectures, and although there is some overlap in associated gene properties there are marked differences as well. Combining different phenotypes into a single GSA is therefore likely to lead to drastically different results.

Comparison

Of the six recent blood pressure GWASs[10-15], all but Kraja et al.[15] performed GSA. In Ehret et al.[10] analyses were performed on combined SBP and DBP blood pressure loci mixed with additional loci from previous literature, using MAGENTA and DEPICT. There were no significant results reported for MAGENTA or for the DEPICT tissue and cell type annotations, but five significant reconstituted gene sets (FDR with $\alpha = 0.05$) were reported. Although three of those relate directly to the cardiovascular system (*abnormal cardiovascular system physiology*, *prolonged QT interval* and *abnormal vitelline vasculature morphology*), all from the Mammalian Phenotype Ontology[22], there is no obvious overlap with our own results. No significant Gene Ontology[23] gene sets were reported, but as these are reconstituted they would be very different from the Gene Ontology sets analysed in this paper, which by itself can explain this discrepancy.

The phenotypes used in Hoffman et al.[13] were SBP, DBP and PP, though these were again combined for the GSA. DAVID was used for the analysis, and 26 significant associations were found

(FDR with α = 0.05). The majority of these were UniProt[24] keywords, which do not directly map to Gene Ontology terms. There is some similarity in the associations of *nucleotide-binding* and *cytoskeleton* with results from our analysis. Four Gene Ontology molecular function and cell component gene sets were found, but these were all very general sets (*membrane*, *centrosome*, *protein binding* and *vacuolar membrane*) with no clear overlap with our findings.

Surendran et al.[12] used four phenotypes (SBP, DBP, PP and HT), which were analysed separately in a MAGENTA analysis. A total of eleven associations were found (FDR with α = 0.05, and additionally requiring that $p < 0.01$), of which six for DBP, three for HT, two for SBP and none for PP. The annotation used largely consisted of canonical pathways and Gene Ontology was not included. Some general similarities were found in the form of *RNA polymerase I promoter clearance* (DBP) and *triacylglyceride biosynthesis* (DBP, HT) associations, somewhat reminiscent of our own associations involving RNA polymerase promoter II as well as biosynthetic processes related to nucleoside phosphates and purine-containing compounds.

For Warren et al.[14] the phenotypes (SBP, DBP and PP) were again combined, and were analysed using DEPICT, FORGE and IPA. The data used was an earlier release of UK Biobank data, and therefore partly overlaps with our own. DEPICT was used to analyse tissue and cell type specific expression, showed involvement of both cardiovascular and urogenital tissues, including arteries, heart and uterus, similar to our own results. Several tissues from the digestive system were also found which overlaps with PP results retained in step two from our own analysis, which included associations for colon and esophagus (these were discarded in step three, as they were entirely explained by the cardiovascular and reproductive system associations).

The FORGE analysis was also for tissue and cell type specific variables but based on Dnase I hypersensitivity sites rather than expression. For the tissues this analysis primarily found cardiovascular involvement, with no clear evidence for urogenital/reproductive system or digestive system tissues. For the IPA gene set analysis the annotation again did not overlap well with our own and is difficult to compare, though the canonical pathways found showed a general theme of cardiovascular involvement.

In Liu et al.[11] all five of the blood pressure phenotypes were used, though again collapsed into general blood pressure associations for the GSA. Analysis was performed using GeneGO and a total of 24 significant biological process gene sets were found (FDR with α = 0.05). Because the annotation was used in these analyses as well these results are much easier to compare to our own, and there is a marked similarity between them. Many of the GO terms found are the same or closely related to those found in our analyses, with several associations relating to (smooth) muscle cells, circulation and cell signaling/communication, as well as for *regulation of blood pressure* and *regulation of urine volume*.

Some of the GeneGO associations are also the same as or similar to associations that were found in step one of our analyses but were later discarded as they were explained by those of other gene properties. In this case the same signal is found, but using our conditional analyses this could be attributed to a different source. Examples of these are the *regulation of blood circulation* and *regulation of system process* sets (initially significant for DBP in our analyses, but found to be confounded by tissue-specific expression), and the *positive regulation of insulin-like growth factor receptor* set reminiscent of the *insulin like growth factor binding* molecular function set initially significant for PP in our analyses.

SUPPLEMENTARY REFERENCES

(1) Sudlow, C. et al. UK Biobank: an open access resource for identifying the causes of a wide range of complex diseases of middle and old age. *PLoS Med.* **12,** e1001779 (2015).
(2) UK10K Consortium et al. The UK10K project identifies rare variants in health and disease. *Nature* **526,** 82-90 (2015).
(3) 1000 Genomes Project Consortium et al. A global reference for human genetic variation. *Nature* **526,** 68-74 (2015).
(4) McCarthy, S. et al. A reference panel of 64,976 haplotypes for genotype imputation. *Nat. Genet.* **48,** 1279-1283 (2016).
(5) Chang, C.C. et al. Second-generation PLINK: rising to the challenge of larger and richer datasets. *Gigascience* **4,** 7 (2015).
(6) Patterson, N., Price, A.L. & Reich, D. Population structure and eigenanalysis. *PLoS Genet.* **2,** e190 (2006).
(7) Abraham, G., Qiu Y. & Inouye, M. FlashPCA2: principal component analysis of Biobank-scale genotype datasets. *Bioinformatics* **33,** 2776-2778 (2017).
(8) Goeman, J.J. & Solari, A. Multiple hypothesis testing in genomics. *Stat. Med.* **33,** 1946-1978 (2014).
(9) Wain et al. Genome-wide association study identifies six new loci influence pulse pressure and mean arterial pressure. *Nat. Genet.* **43,** 1005-1011.
(10) Ehret, G.B. et al. The genetics of blood pressure regulation and its target organs from association studies in 342,415 individuals. *Nat. Genet*. **48,** 1171-1184 (2016).
(11) Liu, C. et al. Meta-analysis identifies common and rare variants influencing blood pressure and overlapping with metabolic trait loci. *Nat. Genet.* **48,** 1162-1170 (2016).
(12) Surendran, P. et al. Trans-ancestry meta-analyses identify rare and common variants associated with blood pressure and hypertension. *Nat. Genet.* **48,** 1151-1161 (2016).
(13) Hoffmann, T.J. et al. Genome-wide association analyses using electronic health records identify new loci influencing blood pressure variation. *Nat. Genet.* **49,** 54-64 (2017).
(14) Warren, H.R. et al. Genome-wide association analysis identifies novel blood pressure loci and offers biological insights into cardiovascular risk. *Nat. Genet.* **49,** 403-415 (2017).
(15) Kraja, A.T. et al. New blood pressure-associated loci identified in meta-analyses of 475 000 individuals. *Circ. Cardiovasc. Genet.* **10,** e001778 (2017).
(16) Segrè, A.V. et al. Common inherited variation in mitochondrial genes is not enriched for associations with type 2 diabetes or related glycemic traits. *PLoS Genet.* **6,** e1001058 (2010).
(17) Huang, W., Sherman, B.T. & Lempicki, R.A. Systematic and integrative analysis of large gene lists using DAVID bioinformatics resources. *Nat. Protoc.* **4,** 44-57 (2009).
(18) Krämer, A., Green, J., Pollard, J. & Tugendreich, S. Causal analysis approaches in Ingenuity Pathway Analysis. *Bioinformatics* **30,** 523-530 (2014).
(19) Dunham, I., Kulesha, E., Iotchkova, V., Morganella, S. & Birney, E. FORGE: a tool to discover cell specific enrichments of GWAS associated SNPs in regulatory regions. *F1000Res*. **4,** 18 (2015).
(20) Pers, T.H. et al. Biological interpretation of genome-wide association studies using predicted gene functions. *Nat. Commun.* **6,** 5890 (2015).
(21) De Leeuw, C.A., Neale, B.M., Heskes, T. & Posthuma, D. The statistical properties of gene-set analysis. *Nat. Rev. Genet.* **17,** 353-364 (2016).
(22) Jupp, S. et al. A new ontology lookup service at EMBL-EBI. In *Proceedings of SWAT4LS International Conference 2015* (eds Malone, J. et al.) (2015).
(23) The Gene Ontology Consortium. Gene Ontology Consortium: going forward. *Nucl. Acids Res.* **43,** D1049-D1056 (2015).
(24) The UniProt Consortium. UniProt: the universal protein knowledgebase. *Nucl. Acids Res.* **45,** D158-D169 (2017).

Supplementary Figures

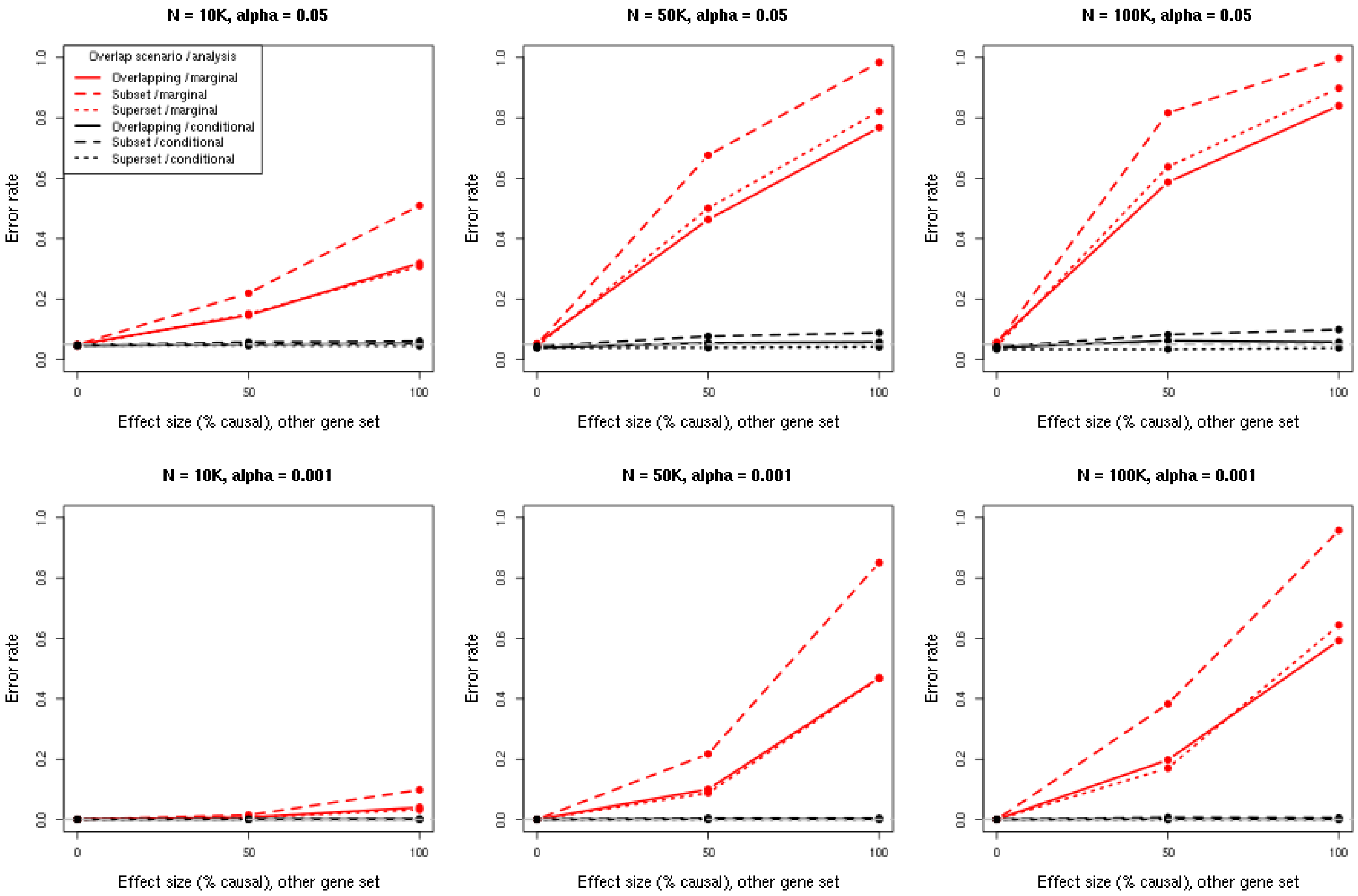

Supplementary Figure 1. Simulation results for the conditional analysis scenario. Gene sets were simulated overlapping with another gene set that was assigned an effect, and were tested using regular GSA and when conditioning on the other set. Type 1 error rates were computed at different significance thresholds, aggregating across ten sets and 1,000 replicates per set for each condition. Results in the plots are also aggregated across conditions over varying gene-set size and overlap parameters. The gray dashed line indicates the nominal type 1 error rate. The gene set is found to be detected at a highly inflated rate when testing the marginal association, but not in the conditional model. Slight deviations from the nominal error rate for the conditional analysis can be seen as well, due to the simulation scenario being only an approximation of the conditional null model.

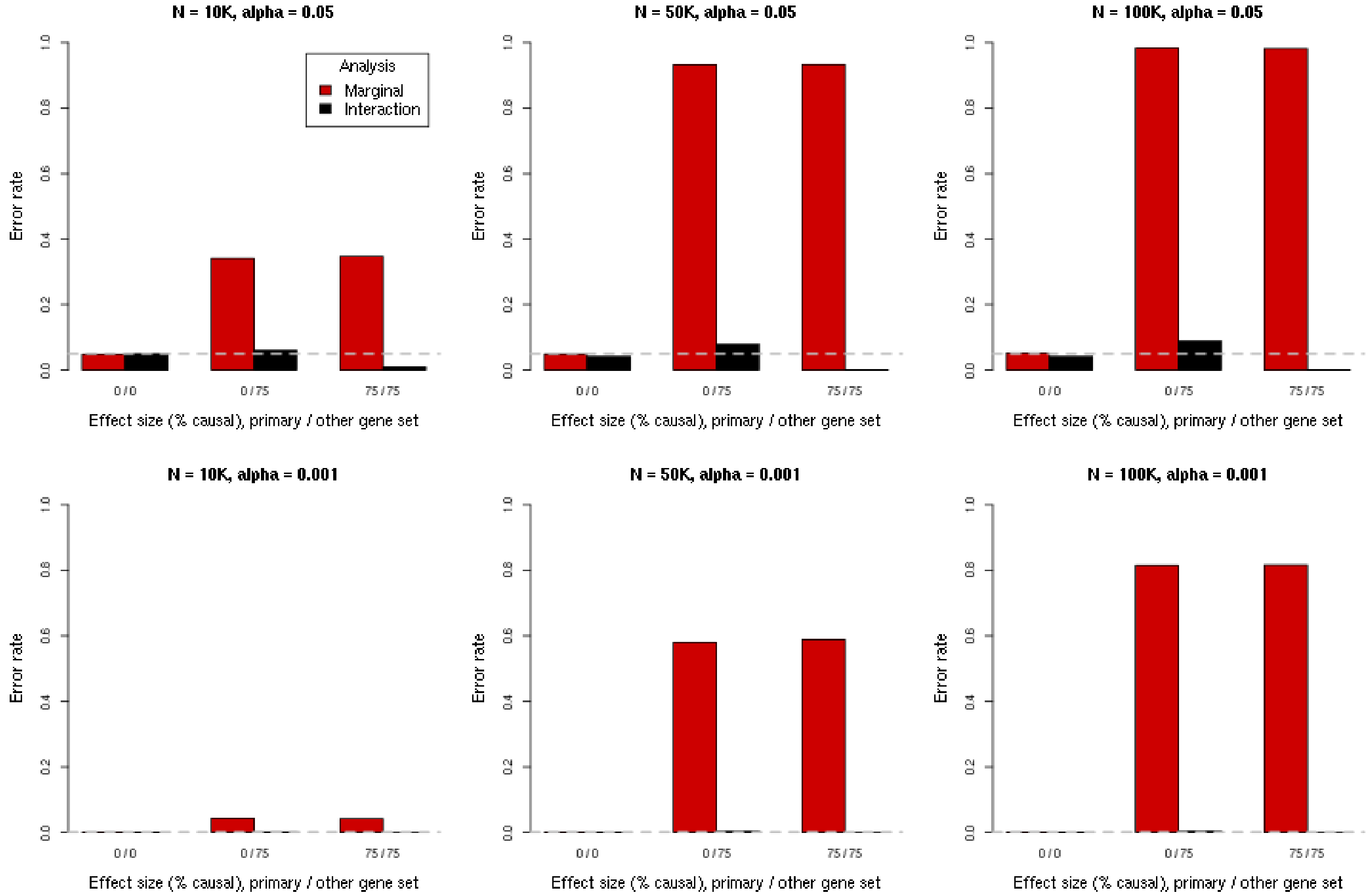

Supplementary Figure 2. Simulation results for the interaction analysis scenario. Overlapping gene sets were generated with an assigned main effect, and the interaction between the two sets was then analysed. The interaction was both tested as a gene set by itself (containing all genes present in both of the gene sets) using regular (marginal) GSA, and when using the full interaction model. Type 1 error rates were computed at different significance thresholds, aggregating across ten interaction sets and 1,000 replicates per interaction set for each condition. The gray dashed line indicates the nominal type 1 error rate. The interaction set is found to be detected at a highly inflated rate when testing its marginal association, but not in the interaction model. Slight deviations from the nominal error rate for the interaction analysis can be seen as well, due to the simulation scenario being only an approximation of the interaction null model.

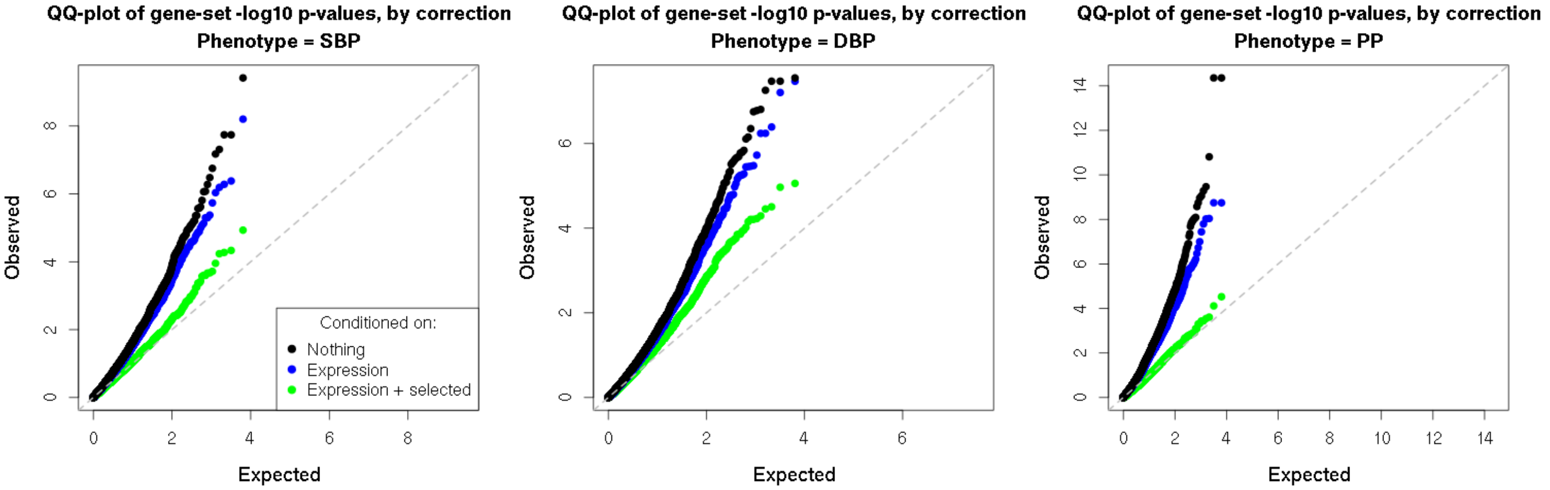

Supplementary Figure 3. QQ-plots of marginal and conditional gene-set -log10 p-values. Results are either marginal associations conditioned on nothing (analysis step 1), conditioned on general confounders (overall expression, top tissue-specific expression levels, miRNA target gene status (miRNA target sets only); analysis step 2), or conditioned on general confounders as well as the gene sets that were significant and retained for that phenotype.

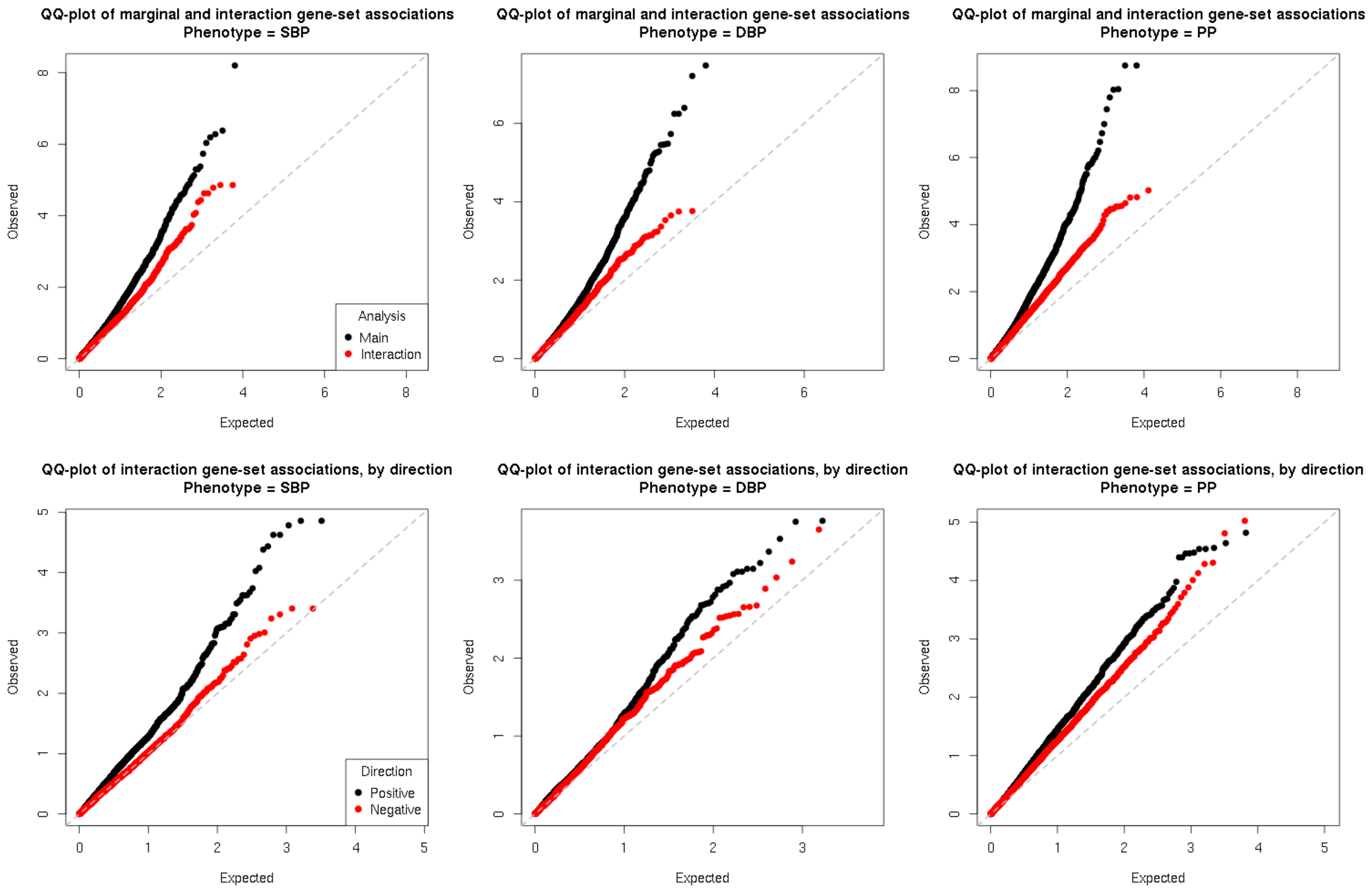

Supplementary Figure 4. QQ-plots of gene set by gene set interaction p-values from exploratory interaction analysis. (Top row) Contrast of interaction QQ-plot with marginal association QQ-plot. Marginal associations are from step 2 in the analysis, conditioned on general confounders. (Bottom row) QQ-plot of interaction p-values split by the direction of the interaction effect.

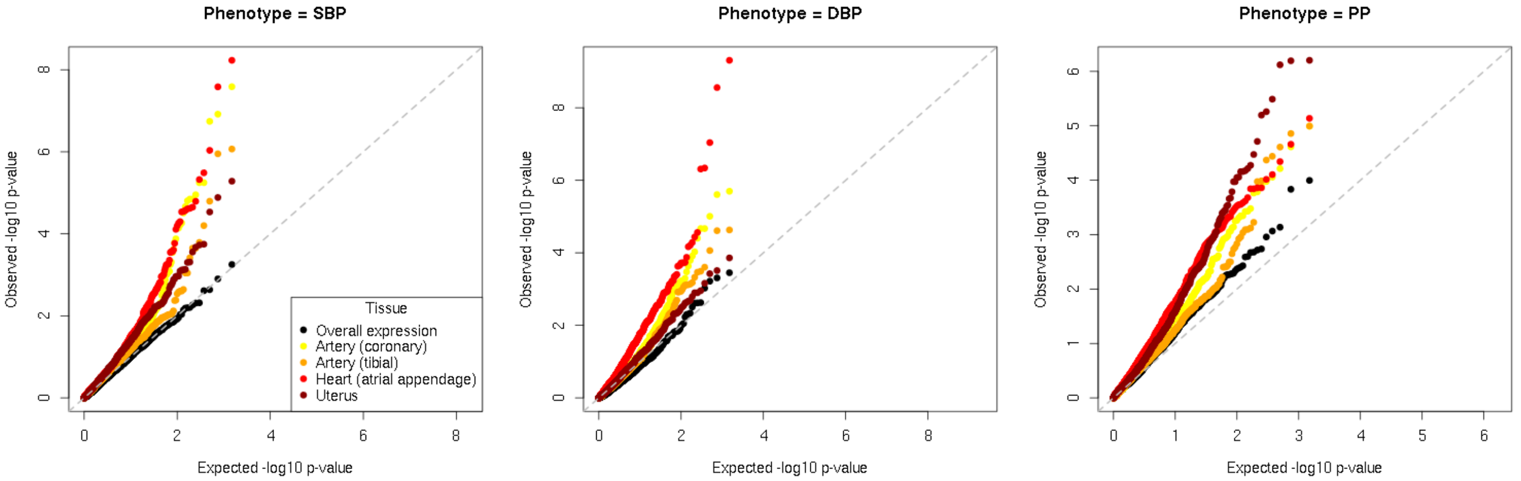

Supplementary Figure 5. QQ-plots of tissue expression by gene set interaction p-values, by tissue. For each tissue, 1,495 interactions were tested. In the analyses for the four specific tissues, the interaction of the gene set with overall expression was also included as a covariate.

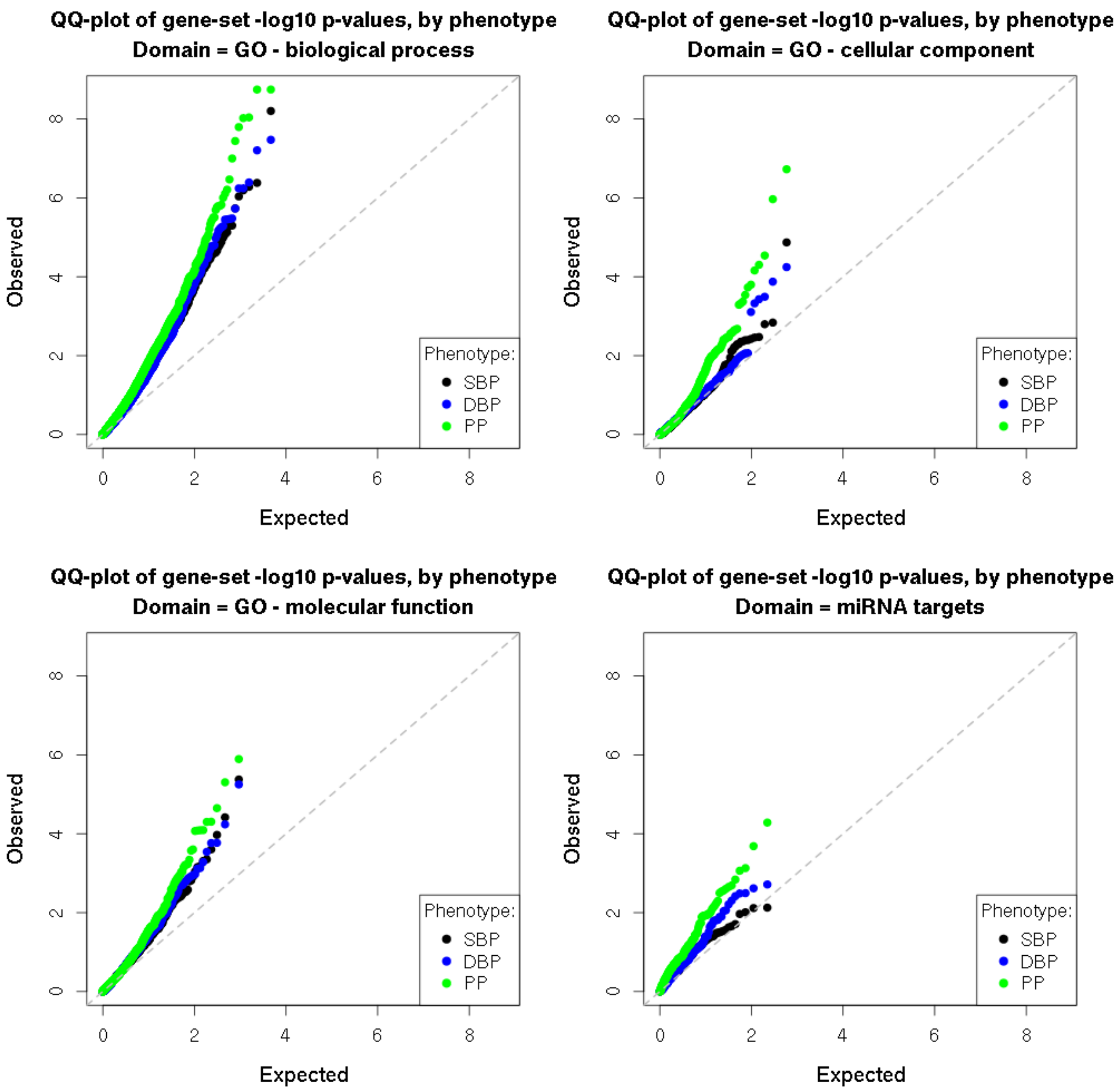

Supplementary Figure 6. QQ-plots of gene-set -log10 p-values by domain. Results are conditional on general confounders (analysis step 2). The axis scale is kept identical across plots, to facilitate comparison.

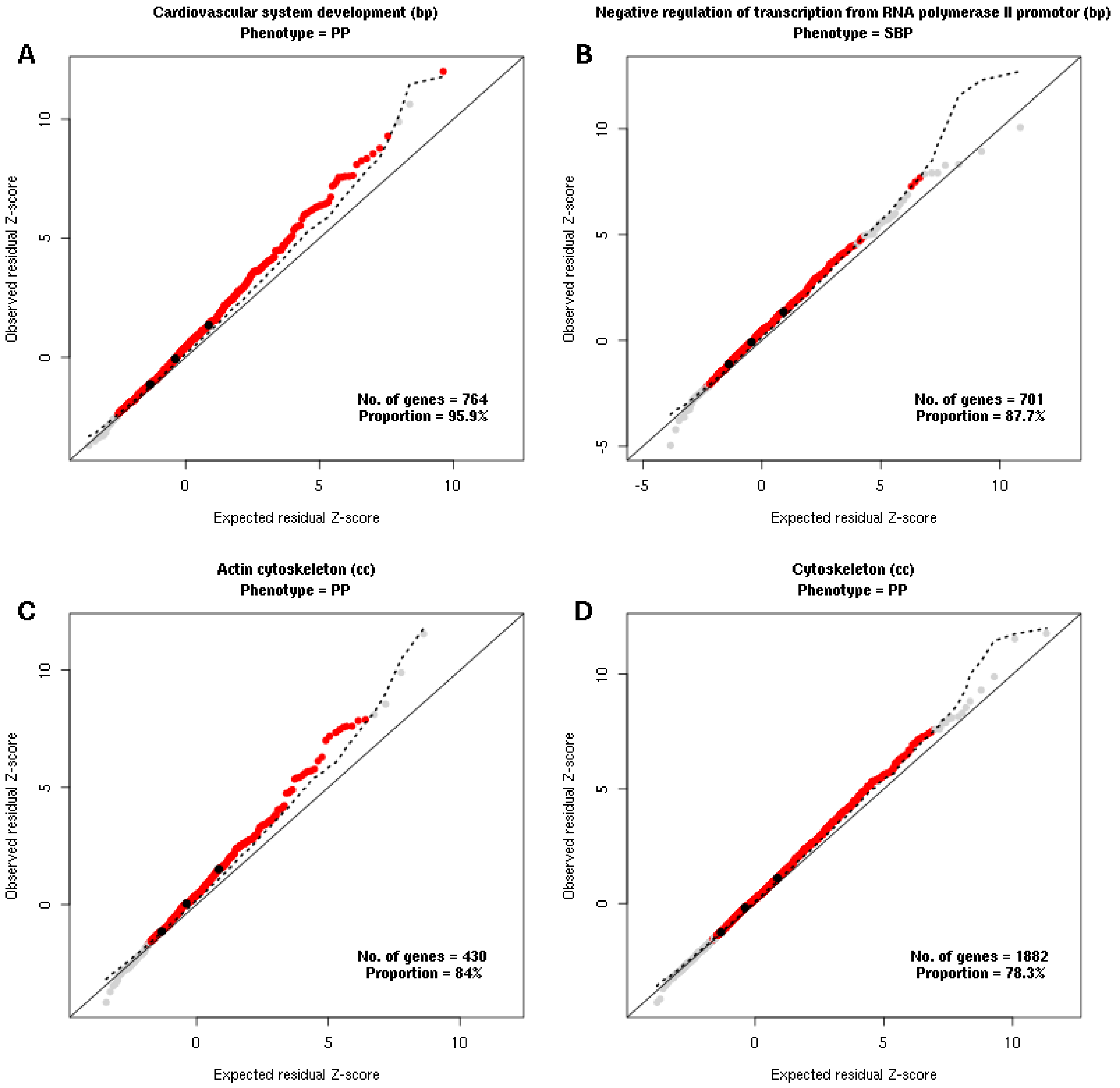

Supplementary Figure 7. Gene-set specific QQ-plots for flagged gene sets, of Z-scores of genes in the gene set. Plotted are the residualised Z-scores from the null model for each gene set, with the expected values based on the quantiles across all genes in the data. The black points denote the 25th, 50th and 75th percentile. The dashed black line is a one-sided (upper) 95% confidence band. Genes are colored red if they exceed the confidence band, and grey otherwise. The proportion given in each plot is the proportion of genes in the set exceeding the confidence band. (A) This gene set was not flagged, but is included for comparison. There is an early and consistent deflection from the plot diagonal, almost all genes fall outside the confidence band and from the median upwards also clearly diverge from the confidence band by a reasonable margin. The lower tail of 'grey' genes is quite small, and stays close to the plot diagonal. (B-D) For these gene sets the proportion is lower and the plot stays closer to the confidence band (especially B and D). The lower grey tail is larger and dips down below the diagonal more strongly. These issues are worth taking into account, but also not deeply problematic.

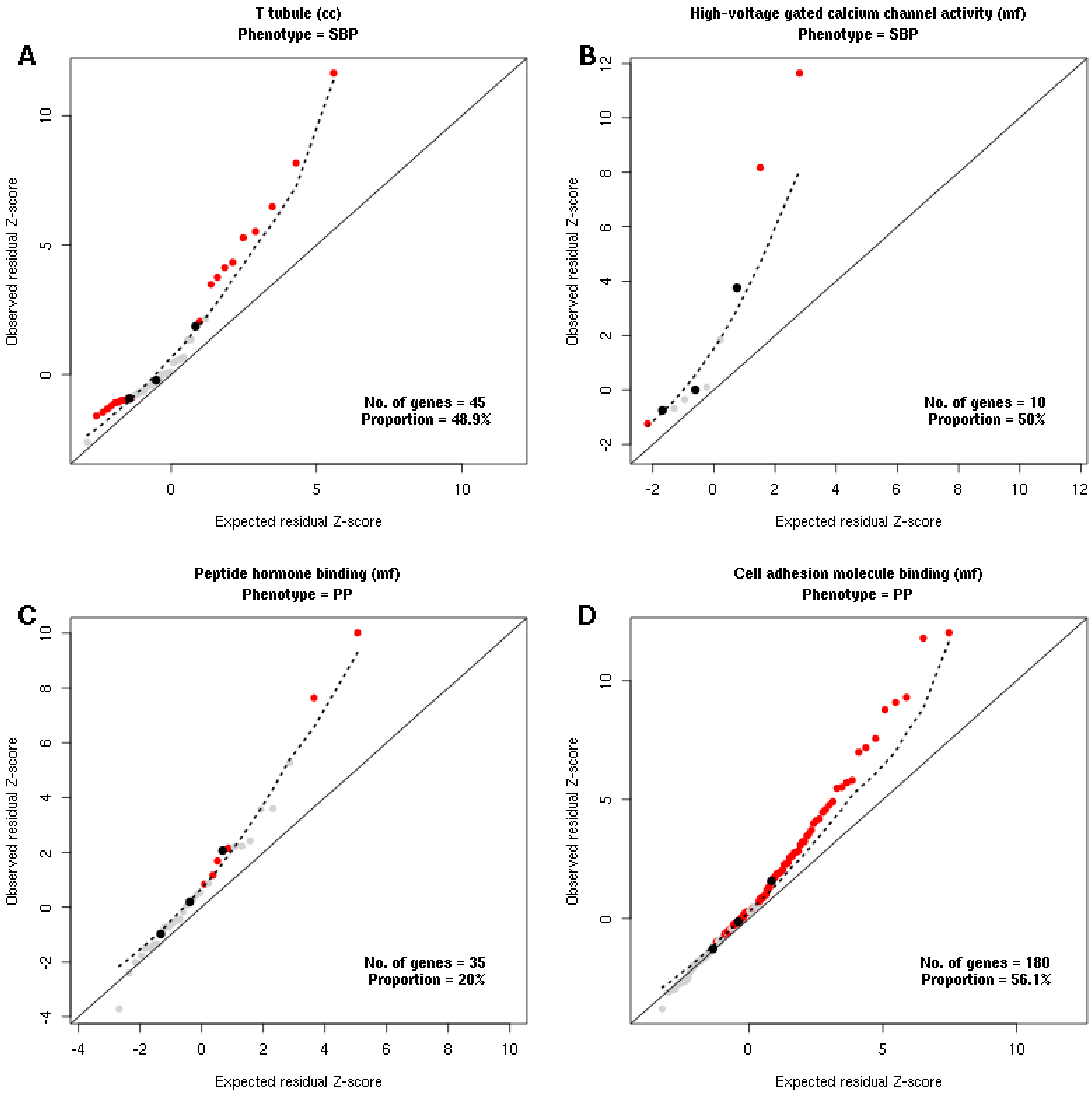

Supplementary Figure 8. Gene-set specific QQ-plots for flagged gene sets, of Z-scores of genes in the gene set. Plotted are the residualised Z-scores from the null model for each gene set, with the expected values based on the quantiles across all genes in the data. The black points denote the 25th, 50th and 75th percentile. The dashed black line is a one-sided (upper) 95% confidence band. Genes are colored red if they exceed the confidence band, and grey otherwise. The proportion given in each plot is the proportion of genes in the set exceeding the confidence band. (A) The middle of the QQ-plot is unusually close to te plot diagonal, this may be the result of this set overlapping with possibly multiple other more directly involved gene properties. (B) Half of the genes are quite close to the plot diagonal, whereas the top two genes strongly exceed the confidence band. Given the small size of this set it is likely that its association is largely caused by those two outlier genes. The set is therefore discarded. (C) This QQ-plot is unusual, in that most of the genes are inside the confidence band. They are consistently above the plot diagonal however, excepting one gene, and as such there does not appear to be much direct cause for concern. (D) For this set there is a long grey tail and the proportion of genes outside the confidence band is not that high. The plot starts to deflect much more quickly from the diagonal about halfway through as well. It is likely that this gene set is confounded by another unknown gene property, causing a partitioning of relevant and irrelevant genes in this set. The association for this set should probably not be interpreted. As it is reasonably strong it can be useful to retain it however, in order to track down the underlying association causing the (likely) confounding.

SUPPLEMENTARY TABLES

Supplementary Table 1. Marginally significant tissue-specific expression variables discarded in step 3

		P-value		
**Tissue**	**Phenotype**	**Marginal**	**Conditional**	**Primary overlap**
Adipose (subcutaneous)	PP	1.41e-5	0.405	Artery
Cervix (ectocervix)	PP	4.74e-5	0.429	Reproductive
Colon (sigmoid)	PP	1.76e-6	0.154	Artery/reproductive
Esophagus (gastroesophageal junction)	PP	4.64e-7	0.406	Artery/reproductive
Esophagus (muscularis)	PP	1.84e-6	0.394	Artery/reproductive
Fallopian tube	PP	2.61e-5	0.473	Reproductive
Heart (left ventricle)	PP	0.000150	0.611	Heart

The marginal p-values are from step 2, corrected for overall expression; the conditional p-values are additionally conditioned on the seven retained tissues listed in Table 2. The 'Primary overlap' column indicates which of the three clusters of tissues the tissue association most strongly overlaps with (Reproductive refers to the cluster of uterus, ovary and endocervix). Associations already discarded in step 2 are omitted from this table as this covers almost all associations; these can be found in Supplementary Table 9 (in the Supplementary Data).

Supplementary Table 2. Marginally significant gene sets that were discarded in the extended analysis

Gene set	No. of genes	Phenotype	P-value Step 1	P-value Step 2	Reason for discarding
GO - biological process					
Anatomical structure formation involved in morphogenesis	931	PP	8.82e-9	7.83e-7	Overlap
Blood vessel morphogenesis	355	PP	4.33e-8	2.03e-5	Confounding
Cardiac muscle cell differentiation	73	SBP	4.90e-8	5.25e-7	Overlap
Cardiac muscle cell differentiation	73	PP	8.79e-10	3.41e-7	Overlap
Cardiac muscle tissue development	138	PP	1.09e-9	1.59e-6	Overlap
Cardiocyte differentiation	95	DBP	1.56e-7	3.52e-6	Confounding
Cardiovascular system development	764	SBP	1.82e-8	5.05e-6	Confounding
Cardiovascular system development	764	DBP	3.36e-8	1.70e-5	Confounding
CGMP metabolic process	21	DBP	1.45e-6	3.32e-6	Confounding
Circulatory system development	764	SBP	1.82e-8	5.05e-6	Confounding
Circulatory system development	764	DBP	3.36e-8	1.70e-5	Confounding
Circulatory system process	351	DBP	7.80e-7	6.82e-6	Confounding
Endocardial cushion development	32	PP	7.80e-7	1.01e-5	Confounding
Heart development	456	PP	3.41e-10	1.61e-6	Overlap
Mesenchymal cell differentiation	131	PP	3.78e-7	2.02e-6	Overlap
Muscle cell differentiation	232	SBP	8.63e-7	1.01e-5	Confounding
Muscle cell differentiation	232	PP	1.57e-6	0.000181	Confounding
Negative regulation of cell aging	17	PP	7.07e-7	1.50e-6	Overlap
Negative regulation of cell proliferation	618	PP	1.23e-7	4.69e-6	Confounding
Negative regulation of transcription from RNA polymerase II promoter	701	PP	8.21e-7	1.90e-5	Confounding
Organ morphogenesis	818	PP	5.59e-8	3.12e-6	Confounding
Positive regulation of gene expression	1662	PP	1.53e-8	1.72e-6	Overlap
Positive regulation of transcription from RNA polymerase II promoter	967	PP	1.03e-6	1.71e-5	Confounding
Regulation of blood circulation	285	DBP	1.60e-6	8.85e-6	Confounding
Regulation of cell proliferation	1428	PP	4.34e-7	1.42e-5	Confounding
Regulation of cellular component movement	745	PP	1.94e-7	1.16e-5	Confounding
Regulation of system process	493	DBP	7.79e-7	3.56e-6	Confounding
Regulation of transcription from RNA polymerase II promoter	1682	SBP	1.77e-7	7.41e-6	Confounding
Regulation of urine volume	19	DBP	1.67e-6	1.88e-6	Overlap
Response to transforming growth factor beta	139	PP	6.66e-7	1.15e-5	Confounding
Skeletal system development	441	PP	2.13e-7	8.44e-6	Confounding
Stem cell differentiation	186	PP	1.22e-6	3.57e-6	Confounding
Tissue morphogenesis	520	PP	1.31e-7	3.12e-6	Confounding
Urogenital system development	291	PP	9.77e-7	9.91e-6	Confounding
Vascular process in circulatory system	158	DBP	2.02e-6	5.55e-6	Confounding
Vasculature development	454	PP	2.65e-9	6.20e-6	Confounding
GO - cellular component					
Actin filament bundle	55	PP	4.45e-6	0.00615	Confounding
Anchoring junction	471	PP	5.45e-7	0.101	Confounding
Cell substrate junction	383	PP	1.09e-5	0.108	Confounding
Cytoskeletal part	1384	PP	1.49e-5	0.0520	Confounding
GO - molecular function					
High voltage gated calcium channel activity	10	SBP	6.19e-6	4.22e-6	QQ - outliers
Insulin-like growth factor binding	15	PP	7.26e-6	4.95e-5	Confounding
Integrin binding	103	PP	1.40e-6	2.24e-5	Confounding
SMAD binding	67	PP	5.32e-6	8.32e-5	Confounding
Transcription factor binding	483	SBP	1.55e-6	3.84e-5	Confounding
Transcription factor binding	483	PP	2.05e-6	8.09e-5	Confounding
miRNA target genes					
miRNA-23A, miRNA-23B	396	PP	1.72e-7	5.21e-5	Confounding
miRNA-221, miRNA-222	136	PP	2.26e-5	0.000207	Confounding
miRNA-495	239	PP	4.14e-5	0.000745	Confounding

In the 'Reason for discarding' column, Confounding refers to the effects of general confounders in step 2 and Overlap to the overlapping with other gene sets as evaluated in step 3.

Supplementary Table 3. Significant but discarded interactions from post-hoc tissue expression by gene set interaction analysis

Tissue	Gene set	Marginal (set)	P-value Interaction	Top 25%	Reason for discarding
SBP					
Artery (coronary)	Lyase activity (MF)	0.881	2.17e-5	0.0830	Outliers
Artery (coronary)	Organophosphate biosynthetic process (BP)	0.062	1.11e-5	0.000611	Outliers
Artery (coronary)	Positive regulation of cyclic nucleotide metabolic process (BP)	0.188	1.57e-5	9.93e-5	Overlap
Artery (coronary)	Positive regulation of nucleotide metabolic process (BP)	0.062	1.42e-5	0.000709	Overlap
Artery (coronary)	Positive regulation of purine nucleotide metabolic process (BP)	0.062	1.42e-5	0.000709	Overlap
Artery (coronary)	Protein alkylation (BP)	0.689	5.66e-6	0.119	Outliers
Artery (coronary)	Protein methylation (BP)	0.689	5.66e-6	0.119	Outliers
Artery (coronary)	S-adenosylmethionine-dependent methyltransferase activity (MF)	0.586	1.21e-7	0.0375	Outliers
Artery (coronary)	Transferase activity transferring one carbon groups (MF)	0.649	3.10e-5	0.189	Outliers
Heart (atrial appendage)	Intracellular signal transduction (BP)	0.0117	2.26e-5	4.41e-5	Overlap
Heart (atrial appendage)	Positive regulation of cyclic nucleotide metabolic process (BP)	0.188	2.84e-5	8.01e-6	Overlap
Heart (atrial appendage)	Positive regulation of homeostatic process (BP)	0.415	2.69e-5	0.0162	Outliers
Heart (atrial appendage)	Positive regulation of hydrolase activity (BP)	0.0405	1.61e-5	0.00160	Outliers
Heart (atrial appendage)	Purine containing compound biosynthetic process (BP)	0.0103	4.75e-6	0.0203	Outliers
Heart (atrial appendage)	Regulation of cyclic nucleotide metabolic process (BP)	0.0872	2.91e-5	0.000214	Overlap
Heart (atrial appendage)	Second messenger mediated signaling (BP)	0.0801	2.24e-5	7.12e-7	Overlap
DBP					
Artery (coronary)	Protein alkylation (BP)	0.285	2.14e-5	0.688	Outliers
Artery (coronary)	Protein methylation (BP)	0.285	2.14e-5	0.688	Outliers
Artery (coronary)	S-adenosylmethionine-dependent methyltransferase activity (MF)	0.452	9.84e-6	0.165	Outliers
Heart (atrial appendage)	miRNA 496 targets	0.538	4.57e-7	0.0622	Outliers
Heart (atrial appendage)	Nucleoside phosphate biosynthetic process (BP)	0.550	4.88e-7	0.000191	Outliers
Heart (atrial appendage)	Purine containing compound biosynthetic process (BP)	0.117	9.13e-8	1.05e-5	Overlap
Heart (atrial appendage)	Secretion (BP)	0.233	2.75e-5	0.0190	Outliers
PP					
Artery (tibial)	Positive regulation of epithelial cell proliferation (BP)	0.00797	2.46e-5	0.0618	Outliers
Artery (tibial)	Regulation of protein kinase B signaling (BP)	0.00854	1.38e-5	0.0195	Outliers
Heart (atrial appendage)	Regulation of blood pressure (BP)	0.0428	7.28e-6	0.000709	Outliers
Uterus	Female sex differentiation (BP)	0.0892	3.24e-6	0.00114	Outliers
Uterus	Male sex differentiation (BP)	0.00137	5.50e-6	5.26e-5	Outliers
Uterus	Positive regulation of cellular amide metabolic process (BP)	0.229	1.94e-5	0.123	Outliers
Uterus	Regulation of reactive oxygen species metabolic process (BP)	0.0232	6.38e-7	0.00163	Outliers
Uterus	Regulation of reproductive process (BP)	0.149	6.36e-6	0.00712	Outliers

Marginal gene-set p-values are from step 2 of the analysis workflow, after correcting for general confounders. The ‘top 25%’ p-values are for the gene set of the 25% genes with the highest residual expression on the tissue, conditioned on the whole set and the tissue expression.

Supplementary Table 4. Significant results using FDR correction for the exploratory gene set interaction analysis

Gene set	Size	Overlap	Marginal p-value	Full model p-values Main	Interaction	Direction
SBP						
Cardiovascular system development (BP)[a]	764	100	5.05e-6	0.00537	2.37e-5	Positive
× Chemical homeostasis (BP)	844		0.000330	0.0492		
Cardiovascular system development (BP)[a]	764	147	5.05e-6	0.0181	1.39e-5	Positive
× Homeostatic process (BP)	1277		0.00832	0.254		
Heart development (BP)	456	51	9.01e-5	0.011	3.68e-5	Positive
× Positive regulation of locomotion (BP)	411		0.0862	0.600		
Regulation of epithelial system development (BP)	277	34	0.000567	0.0723	4.16e-5	Positive
× Epithelial cell development (BP)	178		0.00293	0.295		
Regulation of transcription from RNA polymerase II promotor (BP)	1682	203	7.41e-6	0.00482	1.65e-5	Positive
× Homeostatic process (BP)	1277		0.00832	0.310		
PP						
Cardiovascular system development (BP)[a]	764	100	**1.79e-9**	2.74e-5	2.89e-5	Positive
× Chemical homeostasis (BP)	844		0.000672	0.0836		
Cardiovascular system development (BP)[a]	764	147	**1.79e-9**	2.10e-4	4.02e-5	Positive
× Homeostatic process (BP)	1277		0.000981	0.100		
Cell development (BP)	1369	603	6.92e-5	**1.90e-8**	9.52e-6	Negative
× Cell projection organization	862		0.194	0.00870		
Cell development (BP)	1369	818	6.92e-5	2.58e-6	1.56e-5	Negative
× Neurogenesis (BP)	1348		0.000451	5.86e-5		
Cell proliferation (BP)	645	74	**1.60e-8**	5.51e-5	1.52e-5	Positive
× Regulation of intracellular transport (BP)	596		0.454	0.958		
Mitotic cell cycle (MF)	741	108	0.000128	**1.32e-6**	4.97e-5	Negative
× Cytoskeletal protein binding (BP)	779		0.0241	0.00132		
Negative regulation of developmental process (BP)	732	121	0.000264	0.0705	2.30e-5	Positive
× Double-stranded DNA binding (MF)	757		0.0167	0.475		
Positive regulation of transcription from RNA polymerase II promotor (BP)	967	97	1.71e-5	0.00639	2.77e-5	Positive
× Chemical homeostasis (BP)	844		0.000672	0.0717		
Positive regulation of transcription from RNA polymerase II promotor (BP)	967	151	1.71e-5	0.0182	3.33e-5	Positive
× Homeostatic process (BP)	1277		0.000981	0.0853		
Regulation of cell proliferation (BP)	1428	178	1.42e-5	0.00608	3.43e-5	Positive
× Double-stranded DNA binding (MF)	732		0.0167	0.631		
Regulation of transcription from RNA polymerase II promotor (BP)	1682	203	**9.97e-7**	0.00127	3.45e-5	Positive
× Homeostatic process (BP)	1277		0.000981	0.125		
Regulation of transporter activity (BP)	190	32	3.86e-5	**1.39e-7**	5.22e-5	Negative
× Regulation of calcium-mediated signaling (BP)	72		0.438	0.0388		

Marginal gene-set p-values are from step 2 of the analysis workflow, after correcting for general confounders. Full model interaction p-values are from a two-sided test, main effect p-values are from a one-sided (positive) test. Marginal and main effect p-values printed in **red** are significant under the thresholds applied in step 1.
SBP: systolic blood pressure; PP: pulse pressure; BP: biological process; MF: molecular function
[a] The same interaction exists for *circulatory system development*, which is identical to *cardiovascular system development* and therefore omitted from this table

Supplementary Table 5. Associations of general confounders used in step 2 of the analysis workflow

Gene property	No. of genes	P-value SBP	DBP	PP
Overall expression	-	6.46e-13	1.39e-10	1.44e-13
Artery (coronary) expression	-	0.000959	0.000113	3.14e-10
Artery (tibial) expression	-	0.000646	8.83e-5	1.15e-11
Heart (atrial appendage) expression	-	0.0360	0.00200	2.26e-8
Uterus expression	-	0.000123	4.78e-5	4.03e-8
miRNA target genes	7041	3.31e-7	0.00112	2.36e-7

Except for the overall expression itself, p-values are given for the associations conditional on the overall expression. The *miRNA target genes* gene property was only included as a covariate for analysis of the miRNA target gene sets.

Supplementary Table 6. Replication results for marginal associations of all gene properties in Table 2

		P-value		Significant replication		
**Gene property**	**Phenotype**	**UKB**	**ICBP**	**Nominal**	**Bonf. pheno**	**Bonf. all**
Tissue-specific gene expression						
Artery (aorta)	PP	1.16e-11	0.0853			
Artery (coronary)	DBP	0.000113	0.000424	X	X	X
Artery (coronary)	PP	3.13e-10	0.134			
Artery (tibial)	DBP	8.82e-5	0.00542	X	X	
Artery (tibial)	PP	1.15e-11	0.269			
Cervix (endocervix)	PP	1.58e-6	0.00535	X		
Heart (atrial appendage)	PP	2.26e-8	0.205			
Ovary	PP	5.65e-6	0.0794			
Uterus	SBP	0.000123	0.0789			
Uterus	DBP	4.78e-5	0.00161	X	X	
Uterus	PP	4.03e-8	0.0129	X		
GO - biological process						
Blood vessel remodeling	DBP	5.74e-7	0.0000251	X	X	X
Cardiocyte differentiation	SBP	6.28e-9	0.737			
Cardiocyte differentiation	PP	9.49e-9	0.459			
Cardiovascular system development	PP	1.79e-9	0.0691			
Cell proliferation	PP	1.60e-8	0.0376	X		
CGMP biosynthetic process	DBP	4.06e-7	0.00411	X	X	
Circulatory system development	PP	1.79e-9	0.0691			
Embryonic eye morphogenesis	SBP	4.17e-7	0.496			
Mesenchyme development	PP	9.11e-9	0.00284	X		
Negative regulation of cellular senescence	PP	3.63e-8	0.0109	X		
Negative regulation of smooth muscle cell proliferation	PP	6.20e-7	0.140			
Negative regulation of transcription from RNA polymerase II promotor	SBP	9.22e-7	0.297			
Nitric oxide metabolic process	DBP	6.22e-8	0.241			
Positive regulation of developmental growth	SBP	1.84e-6	0.00604	X		
Positive regulation of urine volume	SBP	6.43e-7	0.0198	X		
Positive regulation of urine volume	DBP	5.72e-7	0.0998			
Reactive oxygen species biosynthetic process	DBP	3.37e-8	0.00236	X	X	
Regulation of smooth muscle cell proliferation	PP	1.00e-7	0.143			
Regulation of transcription from RNA polymerase II promotor	PP	9.97e-7	0.00464	X		
GO - cellular component						
Actin cytoskeleton	PP	1.08e-6	0.369			
Cytoskeleton	PP	1.88e-7	0.685			
T tubule	SBP	1.35e-5	0.0182	X		
GO - molecular function						
Cell adhesion molecule binding	PP	1.28e-6	0.00164	X	X	
Peptide hormone binding	PP	4.96e-6	0.395			
Sequence-specific DNA binding	DBP	5.62e-6	0.264			

UKB p-values are from step 2 of the analysis workflow, after correcting for general confounders. ICBP p-values are computed using the same model and general confounders. Significance for the replication is noted separately for nominal significance, Bonferroni correction for all gene properties in this table per phenotype (Bonf. pheno), and Bonferroni correction for all gene properties in this table across phenotypes (Bonf. all).

Supplementary Table 7. Replication results for interaction associations of all tissue expression by gene set pairs in Table 3

		Interaction p-value		Significant replication		
**Tissue**	**Gene set**	**UKB**	**ICBP**	**Nominal**	**Bonf. pheno**	**Bonf. all**
SBP						
Artery (coronary)	Nucleoside phosphate biosynthetic process (BP)	2.59e-8	0.261			
Artery (coronary)	Purine-containing compound biosynthetic process (BP)	1.81e-7	0.354			
Artery (tibial)	miRNA 145 targets	1.60e-5	0.0474	X		
Artery (tibial)	Nucleoside phosphate biosynthetic process (BP)	8.56e-7	0.251			
Artery (tibial)	Purine-containing compound biosynthetic process (BP)	1.12e-6	0.270			
Heart (atrial appendage)	Positive regulation of catalytic activity (BP)	9.24e-7	0.498			
Heart (atrial appendage)	Positive regulation of molecular function (BP)	3.25e-6	0.302			
Heart (atrial appendage)	Receptor signaling protein activity (MF)	2.39e-5	0.313			
Heart (atrial appendage)	Regulation of blood pressure (BP)	2.61e-8	0.0361	X		
Heart (atrial appendage)	Vascular process in circulatory system (BP)	5.88e-9	0.0562			
Uterus	Development of primary sexual characteristics (BP)	1.30e-5	0.0154	X		
Uterus	Reproductive system development (BP)	2.93e-5	0.133			
Uterus	Sex differentiation (BP)	5.21e-6	0.0451	X		
DBP						
Artery (coronary)	Nucleoside phosphate biosynthetic process (BP)	2.01e-6	0.00254	X	X	
Artery (coronary)	Purine-containing compound biosynthetic process (BP)	2.48e-6	0.0276	X		
Artery (tibial)	Microtubule-based movement (BP)	2.45e-5	0.791			
Artery (tibial)	Microtubule binding (MF)	2.34e-5	0.589			
Heart (atrial appendage)	Regulation of blood pressure (BP)	2.77e-9	0.000861	X	X	X
Heart (atrial appendage)	Vascular process in circulatory system (BP)	4.91e-10	0.000171	X	X	X
PP						
Artery (coronary)	Nucleoside phosphate biosynthetic process (BP)	1.02e-5	0.355			
Artery (coronary)	Purine-containing compound biosynthetic process (BP)	2.44e-5	0.699			
Artery (tibial)	miRNA 145 targets	1.01e-5	0.0118	X		
Heart (atrial appendage)	Cellular response to nitrogen compound (BP)	2.18e-5	0.805			
Uterus	Development of primary sexual characteristics (BP)	7.56e-7	0.00436	X	X	
Uterus	Sex differentiation (BP)	6.26e-7	0.000644	X	X	X

Significance for the replication is noted separately for nominal significance, Bonferroni correction for all interactions in this table per phenotype (Bonf. pheno), and Bonferroni correction for all interactions in this table across phenotypes (Bonf. all).

Supplementary Table 8. Replication results for interaction associations for PP of all gene set by gene set pairs in Table 4

	Interaction p-value		Significant replication	
**Gene set**	**UKB**	**ICBP**	**Nominal**	**Bonf. all**
Cardiovascular system development (BP)[a] × Chemical homeostasis (BP)	1.44e-5	0.0492	X	
Cardiovascular system development (BP)[a] × Homeostatic process (BP)	2.01e-5	0.00772	X	X
Cell adhesion molecule binding (MF) × Glycosaminoglycan binding (MF)	0.000751	0.0142	X	
Cell proliferation (BP) × Regulation of intracellular signal transduction (BP)	0.000204	0.0132	X	
Cell proliferation (BP) × Regulation of intracellular transport (BP)	7.62e-6	0.111		
Regulation of transcription from RNA polymerase II promotor (BP) × Homeostatic process (BP)	1.73e-5	0.0144	X	

Significance for the replication is noted separately for nominal significance and Bonferroni correction for all interactions in this table across phenotypes (Bonf. all).

[a] The same interaction exists for *circulatory system development*, which is identical to *cardiovascular system development* and therefore omitted from this table
