# Supplementary material for: Conditional and interaction gene-set analysis reveals novel functional pathways for blood pressure
Source: Nat Commun. 2018 Sep 14;9:3768. doi: 10.1038/s41467-018-06022-6 (PMC6138636; doi:10.1038/s41467-018-06022-6)
Supplement: Supplementary file 3 — Description of Additional Supplementary Files [file 41467_2018_6022_MOESM3_ESM.pdf]

Description of Additional Supplementary Files

File Name: Supplementary Data 1
Description: Full results of main analyses, interaction analyses and simulations.
